# Enhancing Psychiatry Training Using an Agentic AI Simulated Consultation Tool: Prospective Cohort Study

**DOI:** 10.2196/88580

**Published:** 2026-07-21

**Authors:** Alice Rueda, Huda F Al-Shamali, Zack Cote, Niloy Roy, Reinhard Janssen-Aguilar, Jithin Joseph, Bazen Gashaw Teferra, Mohammad Amin Kamaleddin, Lisa Burback, Olga Winkler, Bill Kapralos, Andrei Torres, Divya Sharma, Sridhar Krishnan, Guillaume Dumas, Andrew Greenshaw, Alexandre Hudon, Sanjeev Sockalingam, Yanbo Zhang, Adam Dubrowski, Venkat Bhat

**Affiliations:** 1 Department of Psychiatry, AI for Mental Health, St. Michael's Hospital, 30 Bond Street, 17-009 Cardinal Carter South, Toronto, ON, Canada, 14163604000, ext 76404; 2 Department of Psychiatry, University of Alberta, Edmonton, AB, Canada; 3 maxSIMhealth Group, Ontario Tech University, Oshawa, ON, Canada; 4 Department of Mathematics and Statistics, York University, Toronto, ON, Canada; 5 Department of Electrical, Computer, and Biomedical Engineering, Toronto Metropolitan University, Toronto, ON, Canada; 6 CHU Sainte-Justine Azrieli Research Center, Université de Montréal, Montréal, QC, Canada; 7 Mila - Quebec Artificial Intelligence Institute, Montréal, QC, Canada; 8 Department of Psychiatry and Addictology, Faculty of Medicine, Université de Montréal, Montréal, QC, Canada; 9 Department of Psychiatry, Institut Universitaire en Santé Mentale de Québec, Montréal, QC, Canada; 10 Centre de Recherche, Institut Universitaire en Santé Mentale de Québec, Montréal, QC, Canada; 11 Department of Psychiatry, Institut National de Psychiatrie Légale Philippe-Pinel, Montréal, QC, Canada; 12 Groupe Interdisciplinaire de Recherche sur la Cognition et le Raisonnement Professionnel (GIRCoPRo), Department of Psychiatry, Université de Montréal, Montréal, QC, Canada; 13 Complex Care and Recovery, Centre for Addiction and Mental Health, Toronto, ON, Canada; 14 Department of Psychiatry, University of Toronto, 250 College Street, Toronto, ON, Canada; 15 Faculty of Health Science, Ontario Tech University, Oshawa, ON, Canada

**Keywords:** psychiatry education, standardized assessment of a clinical encounter report, STACER, agentic AI system, chatbot, consultation skill, psychiatric residents, competence

## Abstract

**Background:**

Canadian psychiatry residents must demonstrate consultation competency, assessed using the standardized assessment of a clinical encounter report (STACER). However, opportunities to practice these skills and receive constructive assessment remain limited in clinical settings.

**Objective:**

This study aimed to evaluate the technical feasibility of an agentic AI system designed to support psychiatry residents’ consultation competence through simulated patient encounters with a patient agent and structured feedback from a rater agent.

**Methods:**

We conducted a two-phase technical feasibility prospective single-arm cohort study of the STACER Agentic System, a large language model–based platform integrating a patient agent and a rater agent. Phase 1 involved automated evaluation of the patient agent using a psychiatrist agent across 227 synthetic major depressive disorder cases. Performance was assessed using DeepEval metrics (correctness, clarity, medical faithfulness, turn relevance, and role adherence) with descriptive statistics and 95% CIs. Phase 2 involved a preliminary user study with 14 convenience-sampled participants: a total of 5 members of the clinical research team and 9 psychiatry residents from the University of Alberta. Participants completed simulated diagnostic interviews and case presentations. Performance was evaluated using STACER-based scoring by the rater agent and 2 psychiatrists. Interrater reliability was assessed using intraclass correlation coefficients (α=.05). Participants rated realism, behavioral consistency, psychiatric nuance, and feedback utility using Likert scales and free-text answers.

**Results:**

The patient agent demonstrated high behavioral (51/56, 91.07%) and symptom fidelity (105/110, 95.45%), with strong automated performance (medical faithfulness mean 0.99, 95% CI 0.99‐1.00; turn relevance 0.99, 95% CI 0.986‐0.992). Participants rated simulations as psychiatrically plausible and diagnostically useful, particularly for depressive symptom representation, although rapport building was moderate (mean 2.78, SD 1.56 to mean 3.00, SD 1.41, out of 5.00) due to limited nonverbal cues. The rater agent generated structured STACER-aligned feedback with high intrarater consistency, especially at the section subtotal level. Interrater reliability with psychiatrists was poor at the item level (intraclass correlation coefficient range=0.25‐0.49) but improved to good-to-excellent agreement at the section level for psychiatry resident sessions (intraclass correlation coefficient range=0.89‐0.93). The rater agent’s scores fell between those of the 2 psychiatrists for the clinical research team and were lower than both human raters for psychiatry residents.

**Conclusions:**

The STACER Agentic System demonstrates the technical feasibility of using agentic AI to simulate psychiatric consultations and deliver STACER-aligned formative feedback. By combining adaptive multiturn psychiatric simulation with competency-based evaluation, it shows promise in supporting cognitive aspects of consultation, though it remains limited in facilitating relational skills such as rapport building. These findings suggest agentic AI could expand scalable, low-risk opportunities for deliberate practice and formative feedback in competency-based psychiatric education. Further controlled studies are needed to evaluate educational effectiveness and integration into residency training.

## Introduction

Psychiatric consultation requires advanced interviewing skills, diagnostic reasoning, and the ability to integrate complex psychosocial information into treatment planning [1,2]. Because the clinical interview and the mental status examination (MSE) remain the cornerstone of psychiatric diagnosis, residents must develop these competencies through repeated, feedback-rich practice [3,4]. Yet, training environments often limit opportunities for sustained supervision and deliberate rehearsal [5,6], leaving residents to encounter challenging cases without sufficient low-stakes practice [7]. Standardized patients offer structured opportunities for experiential learning, but their use is resource-intensive, difficult to scale, and typically limited to major assessments or dedicated simulation events [6,8,9]. As a result, opportunities for iterative practice and formative feedback remain limited, despite their importance for competency development in medical education [10].

In the context of cognitive learning taxonomies such as Bloom's Taxonomy, formative feedback plays a central role in helping learners move beyond basic recall toward higher-order competencies, including analysis, synthesis, and clinical reasoning [11]. For psychiatric interviewing specifically, repeated low-stakes practice enables learners to progress from identifying key symptoms (remember and understand) to formulating differential diagnoses (analyze) and developing treatment plans (evaluate and create). Simulation-based formative feedback therefore supports the deeper cognitive processes required for psychiatric consultation competence by providing structured, iterative opportunities to test and refine reasoning. This mismatch between training needs and available learning opportunities contributes to persistent gaps in consultation skill development.

Digital simulation has emerged as a promising avenue to address this challenge, with AI increasingly enabling applications in diagnosis, risk assessment, and educational evaluation [12,13]. Within this context, virtual patient systems represent a key application of AI-driven simulation in medical education. Early virtual patient systems demonstrated that computer-mediated psychiatric dialogues were feasible and effectively supported specific tasks (eg, obtaining informed consent for antipsychotics) to improve trainee confidence and knowledge in controlled studies [14], as well as enhance diagnostic reasoning [15] and skill acquisition [16]. However, their rule-based logic and fixed response structures constrained realism and adaptability. Case-specific virtual patients, such as Virtual Justina (University of Southern California Institute for Creative Technologies), a virtual adolescent with posttraumatic stress disorder [17], provided helpful structured practice but required extensive manual scripting and lacked the flexibility necessary for diverse, dynamic encounters. As these systems evolve, it is essential that performance measurement for agentic AI systems be aligned with the agent’s expected role. In educational contexts, this means evaluating the system as a training tool rather than as a therapeutic provider. For example, when an AI system is used to help learners practice cognitive behavioral therapy interviewing, its evaluation should focus on whether its responses appropriately model cognitive behavioral therapy–consistent communication and reasoning, rather than implying that the system functions as an actual clinical cognitive behavioral therapy provider [18].

Recent advances in large language models (LLMs) have transformed the capabilities of digital simulation. Transformer-based LLMs [19], trained on terabytes of data, demonstrate improved realism and adaptability in simulating a wide range of patient scenarios relevant to consultation practices [20] and education [21,22]. LLM-generated simulations demonstrate clinically relevant psychiatric exchanges [23-25] and generate automated feedback aligned with expert judgment [26], offering a cost-efficient and scalable alternative to real patients for clinical training. Newer models outperform their predecessors by a significant margin [27]. Tools such as MedSimAI (UCSF [University of California, San Francisco] School of Medicine, Weill Cornell Medicine, Yale School of Medicine, and Ohio State University), which uses GPT-4o to simulate patient encounters and provide immediate formative feedback [28], exemplify the rapid evolution of AI-enabled clinical education. However, current platforms are not designed specifically for psychiatric consultation and often lack validated psychiatric symptom realism, emotional and behavioral nuance, structured MSE-level detail, or alignment with specialty-specific competency frameworks.

In Canada, psychiatry residents’ consultation competence is formally assessed using the structured assessment of a clinical encounter report (STACER) [29], which evaluates performance on the MSE, the DSM-5 (*Diagnostic and Statistical Manual of Mental Disorders* [Fifth Edition])-aligned diagnostic formulation [30], and treatment planning. STACER assessments are infrequent and predominantly summative, limiting opportunities for repeated practice. Kolb's experiential learning cycle [31] underscores that skill development requires iterative cycles of concrete experience, reflective observation, conceptual integration, and active experimentation, conditions difficult to satisfy when assessment opportunities occur only episodically.

To address these limitations and build on our previous works in generative AI and prompt-based reasoning [32,33], we developed the STACER Agentic System (SAS), an LLM-powered simulation and assessment platform designed to support psychiatric consultation training. SAS integrates 2 coordinated AI agents: a patient agent, which simulates realistic multiturn psychiatric interviews, and a rater agent, which produces structured formative feedback aligned with STACER criteria. By unifying simulation and assessment within a single environment, SAS aims to create a scalable pathway for repetitive practice, self-directed learning, and progressive refinement of consultation skills.

The objective of this technical feasibility study was to evaluate SAS’s ability to (1) simulate clinically realistic psychiatric interactions and (2) generate structured, STACER-aligned formative feedback to support the development of consultation competence.

## Methods

### SAS Overview

**Figure 1.**
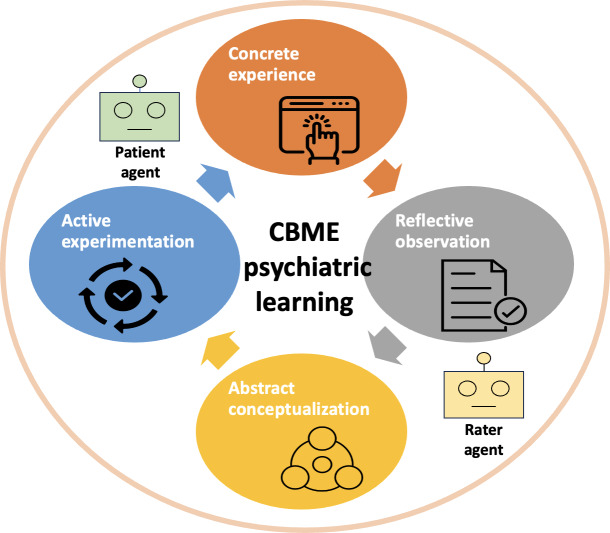
Illustration of how the patient agent and rater agent map onto the four stages of the Kolb experiential learning cycle within a competency-based medical education psychiatric training context. The patient agent supports concrete experience by enabling simulated clinical encounters, while the rater agent contributes to reflective observation through structured assessment. Learners progress to abstract conceptualization by integrating feedback into clinical understanding and to active experimentation by applying these insights in subsequent simulations. CBME: competency-based medical education.

The SAS is an LLM-powered educational platform designed to provide psychiatry residents with structured, simulation-based practice in consultation interviewing. Grounded in principles of simulation-based education [34], the system operationalizes the Kolb experiential learning cycle [31] by coordinating 2 complementary agents: a patient agent, which provides the concrete experience of a simulated psychiatric interview, and a rater agent, which supports reflective observation and abstract conceptualization through structured formative assessment, narrative feedback, and guided cross-examination [10]. Figure 1 illustrates how these agents collectively enact the 4 stages of the Kolb cycle to support competency-based medical education (CBME) [35,36].

The patient agent simulates psychiatric presentations derived from detailed patient profiles, while the rater agent acts as a formative, stabilizing comparator and produces structured formative feedback aligned with the Core of Discipline-STACER form [29]. A verbal chat interface allows residents to ask clarifying questions and engage in reflective dialogue. The system was implemented using LangGraph (LangChain, Inc) [37], an open-source framework that allows for the creation and coordination of fully customizable multiagent systems using graph-based workflows. Patient dialogue is generated using a narrative sense-making process, which draws on research showing that language models naturally use storytelling to make incomplete information feel coherent and realistic [38]. This helps the patient agent produce responses that resemble how real patients naturally describe their experiences and is delivered through integrated audio and speech-to-text tools (supported through ElevenLabs’ AI Audio Application Programming Interface) [39]. For details, please refer to the GitHub repository for the workflow code and the prompt files in Multimedia Appendix 1. Detailed descriptions of the patient agent and the rater agent can be found in Multimedia Appendix 2.

### Study Design

**Figure 2.**
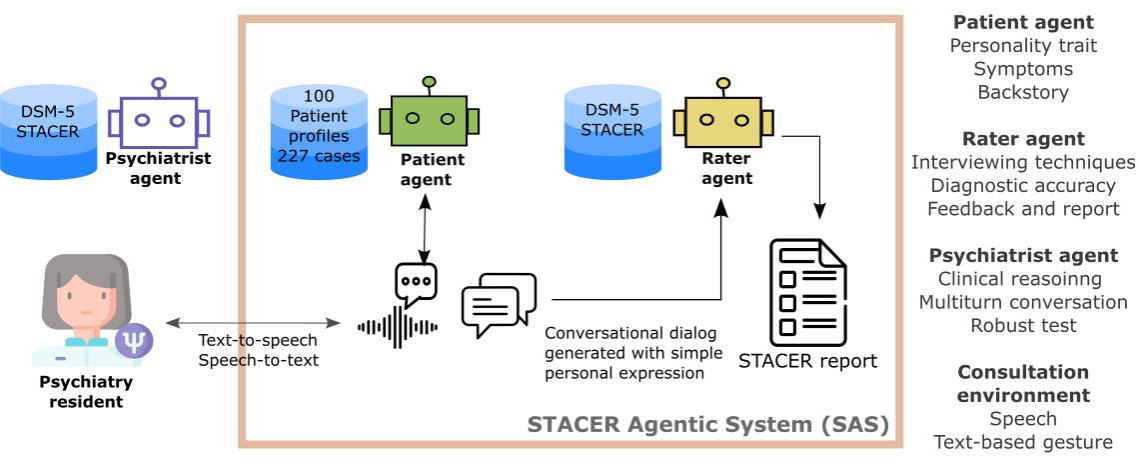
The structured assessment of a clinical encounter report agentic system uses the *DSM-5* (*Diagnostic and Statistical Manual of Mental Disorders* [Fifth Edition]), depression diagnostic criteria, and treatments to provide a simulated environment for psychiatric residents to practice their consultation skills and clinical competency. The patient agent mimics a patient from one of the case studies stored in the patient database, which contains 100 real cases and 227 synthetic cases. The rater agent evaluates the consultation session, provides feedback to the resident, and generates a structured assessment of a clinical encounter report. For technical validation, an external psychiatrist agent was developed to conduct automated multiturn interviews with the patient agent. Grounded in the Core of Discipline-structured assessment of a clinical encounter report expectations, it standardized the elicitation of clinically relevant information across synthetic profiles before human user-testing. STACER: structured assessment of a clinical encounter report.

This study used a two-phase prospective single-arm cohort study design consisting of (1) an automated technical validation and (2) feasibility testing with human participants (Figure 2). To ensure transparent and comprehensive reporting of this LLM-based system, we adhered to the Development, Evaluation, and Assessment of Large Language Models guidelines [40]; the completed Development, Evaluation, and Assessment of Large Language Models checklist is provided in Checklist 1.

### Participants and Setting

Participants included 2 groups: (1) members of the clinical research team and (2) psychiatry residents. The clinical research team consisted of 5 individuals, including 1 student and 4 postdoctoral fellows in psychiatry or psychology. These individuals simulated the role of psychiatry residents during feasibility testing. The second group consisted of 9 psychiatry residents enrolled in the University of Alberta Psychiatry Residency program.

Testing was conducted in 2 settings. Clinical research team members were provided with instructions to complete the simulation sessions in person using a laboratory computer at St. Michael’s Hospital. Psychiatry residents were provided login credentials without further instructions and completed the sessions remotely using the online platform.

No formal sample size calculation was performed, as the study aimed to evaluate feasibility and preliminary system performance rather than to test a hypothesis or detect between-group differences. Participants were recruited using convenience sampling.

### Study Procedure

For technical validation, a psychiatrist agent external to SAS was developed to conduct automated multiturn interviews with the patient agent. The psychiatrist agent, grounded in Core of Discipline-STACER expectations and informed by the *DSM-5*, ensured standardized elicitation of clinically relevant information across all synthetic profiles. Automated testing on the synthetic dataset was performed prior to human user testing.

**Figure 3.**
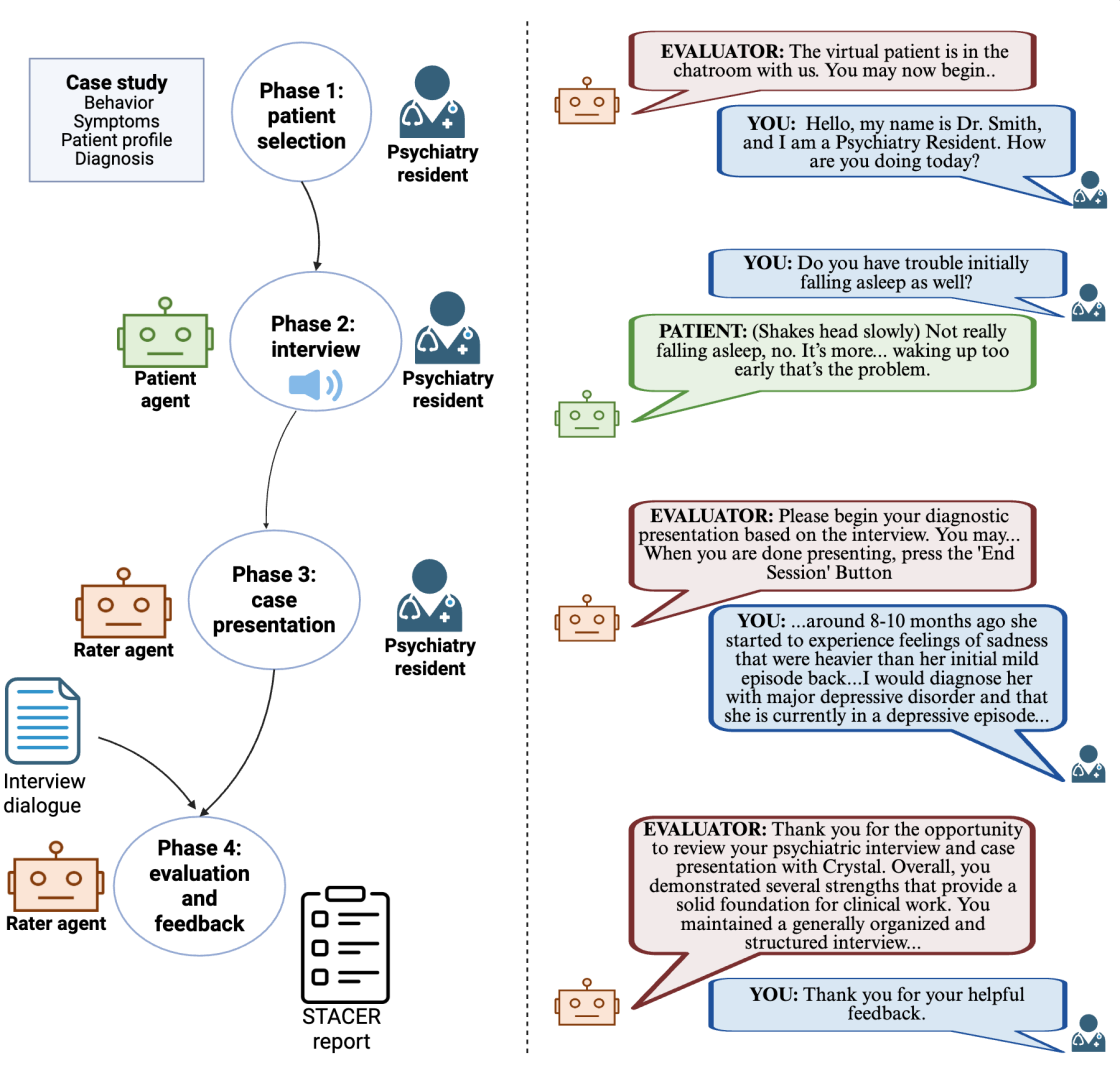
Structured assessment of clinical encounter report agentic system workflow. The left panel shows the four phases: (1) patient selection, (2) simulated interview, (3) case presentation, and (4) assessment and feedback. The right panel illustrates interactions between the psychiatry resident and the structured assessment of clinical encounter report agentic system. The rater agent loads the case and provides an introduction (phase 1). The resident conducts a multi-turn psychiatric interview with the patient agent (phase 2), followed by a case presentation to the rater agent (phase 3). The rater agent then integrates all inputs to generate structured, structured assessment of clinical encounter report agentic system–aligned feedback and a Core of Discipline-Structured assessment of clinical encounter report agentic system assessment, with a chat interface supporting reflection and iterative learning (phase 4). STACER: structured assessment of clinical encounter report.

The feasibility testing unfolded across 4 sequential phases as illustrated in Figure 3: (1) the participant selects a patient profile to interview; (2) a simulated consultation occurs with the psychiatric patient agent (powered by GPT-4.1-mini), mirroring the 55-minute STACER interview; (3) the participant delivers a 20-minute case presentation to the rater agent; and (4) the rater agent (powered by GPT-4.1) combines the case study file, interview dialogue, presentation, and *DSM-5* diagnostic criteria to offer feedback presented in a Core of Discipline-STACER assessment form. A chat interface was provided for participants to converse with the rater agent about the feedback, explore opportunities to extract new knowledge, and engage in active reflection. Simulation results from the 14 participants were manually analyzed and compared with the actual behavior and symptoms of the cases.

Participants rated the patient agent on realism, psychiatric nuance, and rapport building using a Likert-type scale (1=very poor to 5=excellent) and through a free-text option. Two practicing psychiatrists independently reviewed each simulation and rater agent output, providing parallel STACER assessments for comparison.

### Synthetic Benchmark Dataset

Existing datasets are not suited for multiturn psychiatric consultations [41-43], so we developed a comprehensive synthetic benchmark of 227 *DSM-5-Text Revision*–based [44] major depressive disorder cases to systematically evaluate patient agent performance (Table 1). A detailed synthetic case study is provided in Multimedia Appendix 3.

**Table 1.** Synthetic data summary.

	Age, mean (SD)	5 Symptoms	6 Symptoms	7 Symptoms	8 Symptoms	9 Symptoms
Female (n=127)	45.30 (16.96)	63	41	21	2	0
Male (n=100)	45.02 (15.56)	42	36	14	7	1
Total (N=277)	45.74 (16.3)	105	77	35	9	1

### Real-Patient Case Study Dataset

A separate set of 100 manually curated case studies adapted from *DSM-5-Text Revision* Clinical Cases was created for testing. Overall, 5 noncomorbid major depressive disorder cases (5/100, 5%) were selected for feasibility evaluation. Each case included a narrative summary, behavioral description, depressive symptom list, patient profile, and confirmed diagnosis (Multimedia Appendix 3).

### Performance Measure

Performance was evaluated separately for each agent using role-specific metrics, including automated testing for the patient agent using DeepEval (Confident AI); accuracy, clarity, professional tone, medical faithfulness, bias, toxicity, turn relevance, and role adherence), and participant sessions were used in reliability analyses for the rater agent. User feedback questionnaires were used to provide insights into the limitations of the learning experience. The rater agent’s scoring was compared with the psychiatrists’ using the intraclass correlation coefficient (ICC), which was computed using the *psych* package in R (version 4.5.1; The R Foundation for Statistical Computing). Standard interpretive thresholds were applied: ICC <0.50 for poor, 0.50‐0.75 for moderate, 0.75‐0.90 for good, and >0.90 for excellent reliability, respectively [45]. Fidelity of the patient agent’s behavior and symptoms was then analyzed. Full details on performance measures are provided in Multimedia Appendix 4.

### Ethical Considerations

This work was conducted as a preliminary technical feasibility and operational testing study of the SAS prototype prior to future controlled educational evaluation studies. Psychiatry residents and members of the clinical research team voluntarily participated as pilot testers. Participants were informed about the purpose and procedures of the study through email communication and voluntarily chose whether to participate. Given the preliminary operational testing nature of this work, the study focused on prototype functionality and usability assessment rather than formal evaluation of educational or clinical outcomes. Formal research ethics board review was not sought because this work was conducted as preliminary operational and technical feasibility testing of a prototype system prior to future controlled educational evaluation studies.

No personal health information or sensitive participant information was collected. Participant initials were used only for study coordination purposes and were removed prior to analysis. All transcripts, ratings, and feedback were deidentified before reporting to protect participant privacy and confidentiality. No financial compensation or incentives were provided for participation. No identifiable participant information or images are included in the manuscript, figures, or supplementary materials, and no participant assessments or feedback can be linked to individual residents. Future studies designed to formally evaluate learner outcomes, educational effectiveness, or clinical performance will undergo formal research ethics review prior to implementation.

## Results

### Patient Agent Performance

**Figure 4.**
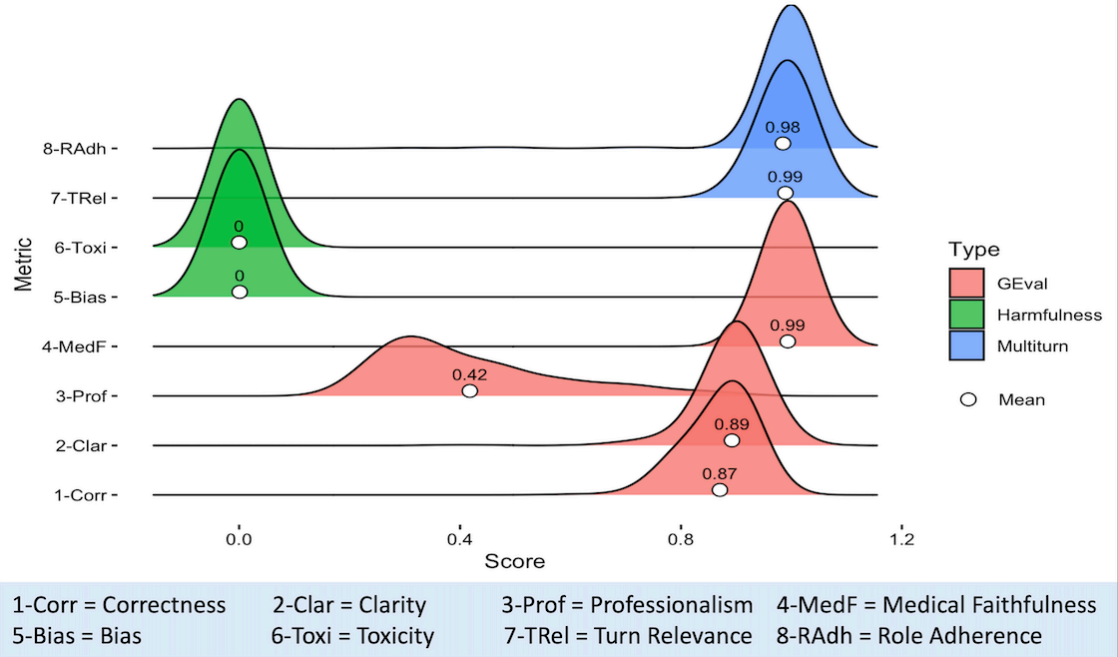
The eight DeepEval performance metrics are colored by the types of metrics, with mean values provided. The density plots indicate that the mean score of the patient agent playing the role of a patient with major depressive disorder using the synthetic dataset: medical faithfulness, turn relevance, and role adherence above 0.98; correctness and clarity above 0.87; and bias and toxicity close to 0. The distribution of scores for the eight metrics with red indicate the GEval measures, green indicates the harmfulness measures, and blue indicates the multiturn measures.

Using the synthesized patient profile and the psychiatrist agent, the Patient Agent’s behavior was validated with the eight DeepEval evaluation metrics. Table 2 and Figure 4 summarize the 8-performance metrics, including mean, SD, 95% CI, token cost, and success rate.

**Table 2.** Patient agent’s performance measure, range 0‐1, using a psychiatrist agent to elicit responses.

	Mean (SD)	95% CI	Total cost (in US $)^a^	Success rate
Correctness^b^	0.87 (0.05)	0.86‐0.88	0.54	1
Clarity^c^	0.89 (0.06)	0.89‐0.90	0.84	1
Professionalism^d^	0.42 (0.16)	0.40‐0.44	0.85	0.24
Medical faithfulness^e^	0.99 (0.01)	0.99‐1.00	1.04	1
Bias^f^	0.001 (0.01)	0‐0.002	1.69	1
Toxicity^g^	0 (0)	—^h^	1.72	1
Turn relevance^i^	0.99 (0.02)	0.986‐0.992	6.36	1
Role adherence^j^	0.99 (0.10)	0.97‐1.00	1.08	0.98

a Total token cost provided by DeepEval. The total token cost from OpenAI exceeded US $100 due to the more expensive GPT-5 and repeated requests due to a time-out.

b Correctness: overall correctness of the patient profile representation.

c Clarity: clarity of the patient’s expression.

d Professionalism: professional tonality.

e Medical faithfulness: medical faithfulness of the symptom presentation.

f Bias: indication of bias in the utterance.

g Toxicity: amount of toxicity created.

h Not applicable.

i Turn relevance: relevancy between each turn (Patient-to-Psychiatrist and the subsequent Psychiatrist-to-Patient, and vice versa).

j Role adherence: patient role adherence over the multiturn conversation.

### User Experience With the STACER Agentic System

Detailed ratings from participants’ first-session responses are presented in Table 3. Participants indicated that the patient agent effectively expressed depressive symptomatology. However, rapport building was more limited (3/5). Ratings of the patient agent’s ability to convey emotional distress varied between the 2 groups (4.6/5 vs 2.78/5). Using voice and text-based nonverbal cues alone, both the patient and rater agents demonstrated moderate levels of emotional expressiveness.

**Table 3.** Quantitative data on user feedback of the first sessions, rated from 0 (very poorly) to 5 (excellent).

Items	Clinical team (n=5)^a^		Residents (n=9)^b^	
	Mean (SD)	Range	Mean (SD)	Range
Q1^c^. The patient effectively conveyed emotional distress.	4.60 (0.55)	4‐5	2.78 (1.56)	0‐5
Q2. The patient’s responses matched typical depressive symptomatology.	4.80 (0.45)	4‐5	5.00 (0.00)	5‐5
Q3. It was easy to build rapport with the patient.	3.00 (1.41)	1‐4	2.78 (1.56)	0‐5
Q4. The patient’s emotional expressiveness improved my diagnostic reasoning.	3.60 (0.55)	3‐4	3.11 (1.45)	0‐5
Q5. The rater’s emotional expressiveness improved my learning and diagnostic reasoning.	3.60 (0.55)	3‐4	3.33 (1.12)	1‐5

a The tests for the clinical team were conducted using text as an input.

b No instructions were provided to the psychiatry residents that both voice and text were supported. Four residents used text as the input and the rest used voice.

c Q: question.

**Figure 5.**
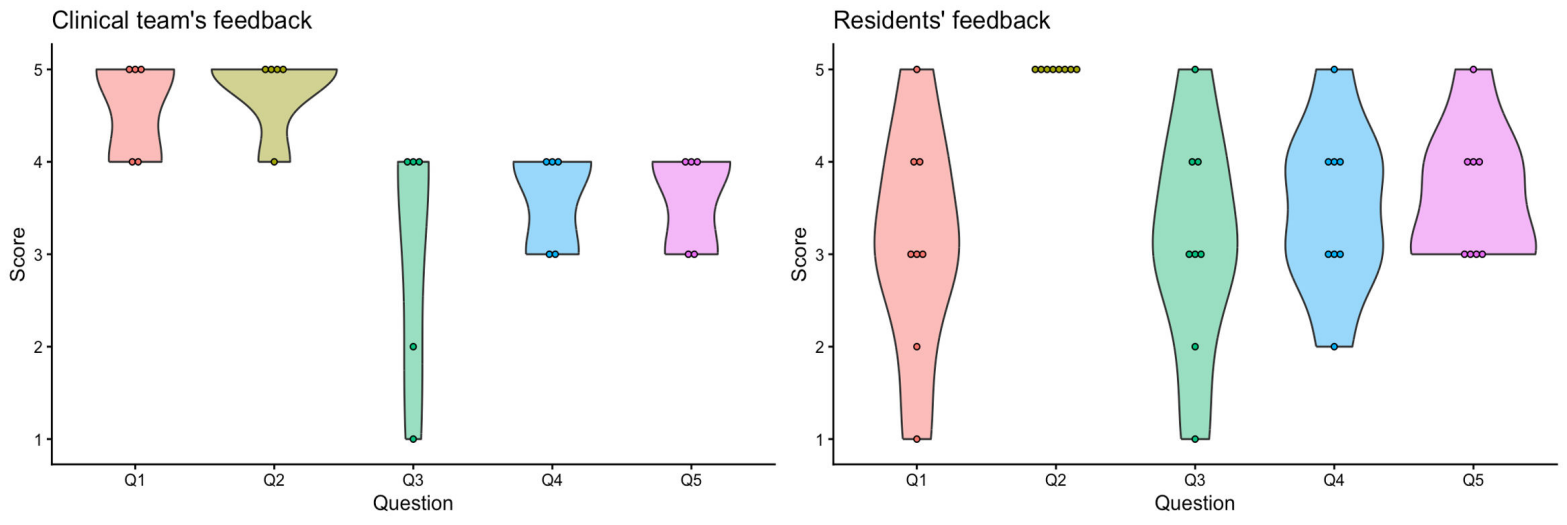
Violin plot of participants’ feedback. Each participant’s response to the survey questions in Table 3 is indicated by a point. (A) Feedback from the clinical research team with instructions provided on the flow of the user interface. (B) Psychiatry residents’ feedback when no instructions were provided. Four of the 9 participants were unaware of the speech capability and used only text. The scores of 0 and 1 were from the text-only participants. Q: question.

Figure 5 illustrates the differences between the 2 participant groups. One of the psychiatry residents assigned 3 scores of “0” and one score of “1,” citing limitations related to typing. The other scores of “1” were given by 1 resident who noted the absence of visual cues and by another who highlighted typing constraints alone.

Free-text feedback indicated that participants found the SAS both informative and educational, not only because of the feedback provided but also due to the ability to ask questions to the rater agent, which enhanced their learning experience and supported their planning. However, the absence of visual cues limited the expression of emotions and hindered rapport building, as facial expressions, body language, and overall appearance are important components of communication.

### Performance Evaluation of the Rater Agent

We measured the consistency of the rater agent at both the item-level scores and the section subtotals. The one-way ICC(1) model, where each target is rated by a different judge and judges are randomly selected, was reported because it yields the most conservative (lowest) correlation estimates. Table 4 summarizes the intrarater reliability of the rater agent across all runs for each recorded session. One of the psychiatry residents (resident 7) did not complete the STACER process, and the session was discarded in the analysis. At the item level, assessments for both groups demonstrated ICCs in the moderate-to-high range (4/5 and 8/8). Reliability improved when item scores were aggregated into section subtotals, with 2 of 5 and 7 of 8 in excellent agreement. Missing data adversely affected reliability estimates, as observed for participants 4 and 5. Resident 7 did not complete the interview and presentation and was excluded from the analysis. The missing data are summarized in Table 4. All missingness belonged to the same items: interrupts politely when required, redirects when required, facilitates organization of disorganized patients, engages patients in a culturally safe manner, attends to timing, and provides a pertinent closing statement. Participant 5 had 3 additional items: nonverbal behavior encourages patients to tell their story, listens attentively, and note taking does not distract from the interview. As these items were not evident and some were impossible to capture from the transcripts, they were replaced with 0.

**Table 4.** Intrarater agent reliability at the item level and section subtotal, with missing scores given 0^a^.

	Missingness (%)	Item-level score			Section subtotal		
		ICC^b^	*F* test (*df*)	95% CI	ICC	*F* test (*df*)	95% CI
Clinical research team (10 runs)							
Participant 1	0	0.80	39.82 (97, 882)	0.74-0.84	0.96	251.65 (4, 45)	0.89-1.00
Participant 2	0	0.89	78.19 (97, 882)	0.85‐0.91	0.96	259.66 (4, 45)	0.89- 1.00
Participant 3	0	0.75	30.31 (97, 882)	0.67- 0.80	0.89	82.44 (4, 45)	0.72- 0.99
Participant 4^c^	1.22	0.45	9.10 (97, 882)	0.37-0.53	0.41	8.00 (4, 45)	0.14-0.87
Participant 5^c^	0.92	0.69	23.20 (97, 882)	0.62-0.75	0.88	72.39 (4, 45)	0.69- 0.98
Psychiatry residents (5 runs)^d^							
Resident 1	3.67	0.80	20.70 (97, 392)	0.74-0.85	0.96	119.38 (4, 20)	0.87-1.00
Resident 2	6.12	0.78	18.46 (97, 392)	0.72-0.83	0.94	80.33 (4, 20)	0.81-0.99
Resident 3	2.45	0.77	18.01 (97, 392)	0.71-0.83	0.94	77.52 (4, 20)	0.81-0.99
Resident 4	3.67	0.59	8.31 (97, 392)	0.51-0.68	0.77	17.46 (4, 20)	0.44-0.97
Resident 5	2.45	0.86	31.20 (97, 392)	0.82- 0.89	0.98	321.59 (4, 20)	0.95-1.00
Resident 6	6.12	0.66	10.73 (97, 392)	0.58-0.74	0.96	122.58 (4, 20)	0.87- 1.00
Resident 8	2.45	0.73	14.37 (97, 392)	0.66-0.79	0.94	85.39 (4, 20)	0.82-0.99
Resident 9	3.76	0.84	27.80 (97, 392)	0.80-0.88	0.992	638.43 (4, 20)	0.97-1.00

a All *P* values are <.001.

b ICC: intraclass correlation coefficient.

c Missing data.

d Resident 7 was not reported, as the interview and case presentation transcripts indicate that the conversation contains only greetings and a short paragraph in the case presentation. The rater agent has given all zero scores to resident 7.

Table 5 summarizes the interrater reliability between the 2 psychiatrists (P1 and P2) and between each psychiatrist and the rater agent. At the item level, all comparisons demonstrated poor agreement, with ICCs below 0.50. The 2 groups exhibited very different results at the section subtotal level. For the clinical team, 1 psychiatrist showed moderate agreement with the rater agent, while the other achieved good agreement. The agreements for the psychiatry residents were much higher, from 0.89 to 0.93, indicating good-to-excellent agreements.

**Table 5.** Inter-rater reliability between the two practicing psychiatrists and the rater agent^a^.

	Item-level score			Section subtotal		
	ICC^b^	*F* test (*df*)	95% CI	ICC	*F* test (*df*)	95% CI
Clinical research team (5 participants, 10 runs)						
P1 versus P2	0.25	1.66 (489, 490)	0.16-0.33	0.42	2.47 (24, 25)	0.05-0.70
P1 versus rater	0.39	2.26 (489, 490)	0.31-0.46	0.75	7.02 (24, 25)	0.52-0.88
P2 versus rater	0.35	2.08 (489, 490)	0.27-0.43	0.58	3.78 (24, 25)	0.26-0.79
Psychiatry residents (8 participants, 5 runs)^c^						
P1 versus P2	0.19	1.48 (775, 776)	0.12-0.26	0.93	28.22 (44, 45)	0.88-0.96
P1 versus rater	0.25	1.67 (775, 776)	0.18-0.31	0.89	18.00 (44, 45)	0.82-0.94
P2 versus rater	0.49	2.92 (775, 776)	0.43-0.54	0.92	24.28 (44, 45)	0.86-0.96

a All *P* values are <.001

b ICC: intraclass correlation coefficient.

c Resident 7 was not reported, as the interview and case presentation transcripts indicate that the conversation contains only greetings and a short paragraph in the case presentation. The rater agent has given all zero scores to Resident 7.

Discrepancies between raters were also evident in overall scoring patterns, especially for the clinical research team. One psychiatrist rated scores 51% higher than the rater agent, whereas the other rated scores 33% lower. The rater agent’s scores consistently fell between those of the 2 psychiatrists. On the other hand, both human raters scored 14% higher than the rater agent for the psychiatry residents.

As shown in Tables4  5, aggregating item scores into section subtotals generally improved agreement. Section-level aggregation provides a broader impression of skill categories rather than focusing on individual items, thereby reducing instability arising from ambiguity or differing interpretations of item definitions.

### Linking Back the Patient Agent’s Behavior and Symptom Expressions

As the participants were given the choice to select the case studies, based only on the case name, the number of behavior traits and symptoms varies (summarized in Table 6). A total of 4 case studies (a-d) were selected by more than 1 tester. Case studies (b) and (c) achieved 100% in behavioral expressions, while case studies (a) and (d) achieved 87.50% (7/8) and 92% (11/12), respectively. Case studies (a), (b), and (d) achieved 100% symptom expression, while case study (c) achieved 88.89% (16/18). Resident 3 selected a Schizophrenia patient profile where behaviors could not be mimicked in the consultation. Resident 9 selected a more complicated comorbid case study. The overall behavioral fidelity across both groups was over 91.07% (51/56), and symptom fidelity reached 95.45% (105/110), consistent with the high medical faithfulness (0.993) from the automated test.

**Table 6.** Patient agent’s behavior and symptom expressions.

Subject	Behavior	Symptoms
Clinical team		
Participant-1^a^	3/4	7/7
Participant-2^a^	4/4	7/7
Participant-3^a^	4/4	9/9
Participant-4^a^	4/4	7/7
Participant-5^a^	4/4	7/7
Overall, score (%)	19/20 (95%)	37/37 (100%)
Residents^b^		
Resident-1^a^	3/3	9/9
Resident-2	4/4	7/7
Resident-3	5/7	6/7
Resident-4^a^	3/4	9/9
Resident-5^a^	3/3	7/9
Resident-6^a^	4/4	9/9
Resident-8	3/4	6/6
Resident-9^c^	6/6	15/17
Overall, score (%)	32/36 (88.89)	68/73 (93.15)

a These are 4 case studies that have been used in the simulation multiple times.

b Resident 7 was not reported, as the interview and case presentation transcripts contain insufficient content for analysis.

c The long list of symptoms is due to comorbidity.

## Discussion

This study evaluated the technical feasibility of an agentic AI platform designed to simulate psychiatric consultations and deliver structured formative feedback aligned with the STACER framework. The findings demonstrate that LLM-driven agents can emulate clinically coherent psychiatric dialogue, support structured feedback, and create an accessible environment for deliberate practice. At the same time, the results highlight specific constraints, including rapport formation and multimodal fidelity, that shape how agentic systems should be incorporated into psychiatric education. These findings suggest that while AI-supported simulation may effectively extend opportunities for experiential learning, there are areas requiring refinement and testing before widespread curricular integration.

### Fidelity and Educational Value of the Patient Agent

The patient agent exhibited high psychiatric fidelity, maintaining diagnostic, behavioral, and linguistic coherence across multiturn interviews. This aligns with evidence that LLM-based virtual patients can support clinically realistic interactions in domains such as communication and history taking [28]. For instance, MedSimAI demonstrated that GPT-4o can sustain coherent exchanges, allowing learners to rehearse clinical questioning strategies [28]. Similarly, Virtual Justina showed that structured, *DSM* (*Diagnostic and Statistical Manual of Mental Disorders*)-based simulations can facilitate practice with sensitive symptom domains, including trauma-related questioning and rapport building [17]. The SAS builds on these foundations by coupling psychiatric realism with adaptive, multiturn dialogue and structured competency mapping aligned to STACER domains, while offering more sustained internal consistency across extended interviews than earlier systems, which were constrained by fixed dialogue trees [17]. The near-perfect turn relevance and role adherence further support its role as a reliable platform for the “concrete experience” stage of experiential learning.

A key finding from both quantitative and qualitative data is that rapport formation was consistently limited (2.78‐3.00 out-of 5 across groups). Rather than a minor usability issue, this represents a defining boundary of current agentic AI systems: while effective for supporting the cognitive components of psychiatric consultation (eg, history taking and diagnostic reasoning), they are less suited for affective and relational skills [17,28,46]. This limitation is further clarified when mapped onto the STACER framework [29]. The patient agent demonstrated strong alignment with domains related to information gathering, diagnostic formulation, and MSE structure; however, lower rapport scores indicate reduced capacity to support domains involving affect, engagement, empathy, and relational skills. These competencies rely on subtle interpersonal and embodied cues, particularly nonverbal communication, that are not fully captured in text- and voice-based interfaces.

These findings are consistent with prior literature indicating that embodied conversational agents vary widely in their use of human communication modalities; although some systems incorporate verbal and nonverbal behaviors, many rely on simpler, less expressive designs [46]. Because advanced multimodal interaction is technically demanding and still seldom evaluated in clinical settings, the extent to which embodied conversational agents can support human-like therapeutic connection remains uncertain. Complementing this, Tay et al [47] found that immersive and augmented reality environments enhance empathy and reduce stigma by enabling learners to experience affective and perceptual dimensions of mental illness. Incorporating audio-prosodic variation, avatar-based facial animation, or sensor-based detection of the learner’s nonverbal cues may partially address this limitation by creating a more ecologically valid simulation of the mental status examination and the relational aspects of psychiatric care [48]. Similarly, any multimodal extension would also need to capture and interpret the resident’s own nonverbal communication, as authentic rapport relies on bidirectional cues [49].

### Performance and Bias in the Rater Agent

The rater agent produced detailed, criterion-referenced feedback and structured STACER scoring, reflecting the growing literature demonstrating that LLM-based evaluators can generate coherent assessments [26,28]. Prior work by Holderried et al [26] showed that GPT-4 achieved “almost perfect” agreement with human raters in history-taking evaluations, while Hicke et al [28] reported that LLM feedback enhanced learner reflection and engagement in simulated patient interactions. In this study, all participants perceived the rater agent’s feedback as specific, actionable, and aligned with expectations for psychiatric assessment. These findings, echoed by Cook et al [23], who observed that LLM-powered virtual patients generated personalized, high-quality performance feedback rated comparably to human evaluators, substantiate the potential of AI-assisted formative assessment in clinical education.

With the use of a more structured and regimented prompt, the rater agent demonstrated improved rating consistency, particularly at the section subtotal level, where agreement ranged from moderate to excellent across both participant groups. Notably, agreement between the rater agent and human raters was higher in the psychiatry resident group than in the clinical research team, suggesting that the system may better approximate expected scoring patterns within its intended learner population. For the clinical research team, the rater agent’s scores consistently fell between those of a more generous and a stricter human rater, indicating a stabilizing, middle-ground evaluation rather than systematic bias. In contrast, for psychiatry residents, both human raters consistently assigned higher scores than the rater agent. This shift, from disagreement in overall scoring for the clinical research team to consistently higher ratings by both psychiatrists for residents, may reflect a favorable bias toward psychiatry residents, potentially influenced by expectations of trainee competence [50]. Blinding raters to participant groups would have helped mitigate this possibility and represents an important consideration for future study design.

Contrary to concerns raised in MedSimAI [28], no evidence of a “supportive” bias was observed. Through iterative prompt engineering, we mitigated several known limitations of LLM-based evaluators, including (1) a predisposition toward overly supportive or affirming judgments [51], (2) overreliance on linguistic polish as a proxy for empathic or clinical competence [52], and (3) the absence of external evaluative pressure that typically constrains human raters [50]. In psychiatric education, such unchecked leniency, while potentially reducing learner anxiety, risks misrepresenting clinical competence and may ultimately diminish the formative value of assessment. To strengthen reliability, future development should incorporate expert-in-the-loop calibration, reinforcement learning informed by clinician judgments, and iterative psychometric validation. Structured prompting strategies, stability constraints, and inconsistency penalties may further reduce variability. Since STACER is used nationally to evaluate readiness for independent practice, any AI-generated scoring must be tightly aligned with established competency standards. Even when used purely for formative purposes, calibration is critical to ensure that learners receive accurate guidance that meaningfully informs their development.

### Alignment With Competency-Based and Experiential Learning

A key contribution of SAS is the way it operationalizes competency-based medical education [35,36] and the Kolb experiential learning cycle [31] within a unified, self-directed, digital ecosystem. Unlike traditional STACER assessments [29], which are episodic and summative, the SAS supports repeated low-stakes practice with immediate feedback, enabling residents to iteratively refine interviewing, diagnostic reasoning, and communication skills. The patient agent provides concrete experience as learners engage in full psychiatric interviews. The rater agent delivers reflective observation and abstract conceptualization through structured narrative feedback, cross-examination questions, and STACER-aligned performance indicators. Reengagement with the patient agent allows for active experimentation, completing the experiential cycle [31]. This sequence mirrors established mechanisms of learning in simulation-based education, where structured debriefing is consistently identified as the primary driver of skill acquisition [53,54]. Cheng et al [53] demonstrated that structured debriefing is an influential instructional component, facilitating reflection, performance analysis, and knowledge transfer to clinical contexts. For example, the Promoting Excellence and Reflective Learning in Simulation framework integrates self-assessment, guided discussion, and directive feedback [54]. The rater agent’s structured questioning and targeted feedback map onto these principles, helping learners identify gaps and refine the interpretive steps that underlie psychiatric consultation competence.

By embedding simulation and assessment within one system, SAS presents a distinct pedagogical affordance: the ability to support adaptive, longitudinal skill development outside of resource-intensive, faculty-led simulation centers. This positions the technology as a complement, not a replacement, to supervised clinical encounters and traditional Objective Structured Clinical Examinations–style assessments [55]. However, these findings should be interpreted cautiously, as the small sample size and open-label feasibility design primarily support conclusions regarding technical feasibility rather than educational effectiveness, thereby limiting generalizability to broader curricular integration.

### Limitations and Future Directions for Educational Implementation

Several limitations must be acknowledged when interpreting these findings. First, phase 1 relied on automated testing using the LLM-as-a-judge paradigm [56,57], enabling high-volume evaluation but limiting the ecological validity of results. Automated benchmarking is useful for characterizing agentic behavior but cannot substitute for human educational outcomes. Second, although psychiatry residents were included, the sample size was small, and variability in interface use (text vs voice) introduced heterogeneity in the user experience, limiting generalizability and precluding conclusions about educational efficacy. Future studies should use randomized controlled designs with larger samples focused on psychiatry residents to evaluate competence development, diagnostic accuracy, and changes in consultation confidence. Third, and most notably, the limited capacity for rapport formation and nonverbal communication directly impacts multiple STACER domains, including affect, engagement, and relational skills. These findings reinforce that current text- and voice-based agentic systems cannot fully replicate the interpersonal complexity of psychiatric encounters. Future development should prioritize multimodal enhancements to better capture both patient and learner nonverbal behaviors and improve ecological validity.

Fourth, the current evaluation of SAS focused mainly on major depressive disorder. A broader evaluation of *DSM-5-Text Revision* diagnostic categories is needed for the system to serve as a comprehensive training tool. Finally, refinement of the rater agent, including calibration to professional standards and psychometric validation, is necessary for improving reliability, fairness, and consistency across learners. Integrating expert-driven feedback loops and controlled prompting strategies may reduce variability in scoring behavior and align the rater agent more closely with human evaluators. To strengthen its educational impact, future iterations of the SAS should also include learning-progress tracking and adaptive difficulty scaling to personalize training trajectories and support longitudinal skill development. Collectively, these limitations inform a clear roadmap for future development: expanding diagnostic breadth, enhancing multimodal realism, increasing participant diversity, and strengthening assessment reliability.

### Conclusion

This technical feasibility prospective cohort study demonstrates that agentic AI systems can simulate clinically coherent psychiatric encounters and generate structured, STACER-aligned formative feedback within a single educational platform. Unlike earlier virtual patient systems that depended on scripted interactions or lacked psychiatry-specific competency assessment, the SAS combines adaptive multiturn psychiatric dialogue with structured evaluation aligned to competency-based medical education frameworks. The findings suggest that the SAS may provide a scalable and accessible approach for supporting deliberate practice, particularly for cognitive aspects of consultation such as history taking and diagnostic reasoning. At the same time, the study highlights an important limitation of current agentic AI systems: reduced capacity to support affective and relational skills, including rapport building, which rely on multimodal and interpersonal cues. By expanding access to repeated low-risk practice and structured feedback, the SAS has the potential to complement traditional supervision and simulation-based training, particularly in resource-constrained educational settings. Although preliminary findings from psychiatry residents are encouraging, the small sample and feasibility design preclude conclusions about educational effectiveness or curricular integration. Future studies should include larger controlled evaluations, multimodal enhancements, and further calibration of the rater agent to strengthen reliability and educational impact.

## Supplementary material

10.2196/88580 Multimedia Appendix 1 Prompts.

10.2196/88580 Multimedia Appendix 2 Detailed description of the STACER Agentic System.

10.2196/88580 Multimedia Appendix 3 Case Study Examples.

10.2196/88580 Multimedia Appendix 4 Performance Measure.

10.2196/88580 Checklist 1 Development, Evaluation, and Assessment of Large Language Models checklist.

## Abbreviations

CBME: competency-based medical education
*DSM*: *Diagnostic and Statistical Manual of Mental Disorders*
*DSM-5*: *Diagnostic and Statistical Manual of Mental Disorders* (Fifth Edition)
ICC: interclass correlation coefficient
LLM: large language model
MSE: mental status examination
SAS: STACER Agentic System
STACER: structured assessment of clinical encounter report
UCSF: University of California, San Francisco
