# Supplementary material for: Enhancing Psychiatry Training Using an Agentic AI Simulated Consultation Tool: Prospective Cohort Study
Source: JMIR Med Educ. 2026 Jul 21;12:e88580. doi: 10.2196/88580 (PMC13387278; doi:10.2196/88580)
Supplement: Multimedia Appendix 2 [file mededu-v12-e88580-s002.docx]

Appendix 1: Agentic System Design

The SAS combines two key components: the Patient Agent assessment and the Rater Agent assessment. The Patient Agent simulated realistic psychiatric symptoms, behaviours, and emotional expressions. As real-time facial expression and body gesture generation were not available, the SAS provided a limited set of facial emotional expression. Text and speech are the main media for conversation where behaviour description, such as psychomotor retardation, was described in text. Trainees interacted with the Patient Agent through a sequential conversation to practice clinical assessment skills. We noted this reduces the MSE scope to exclude assessment of appearance, motor behaviour, psychomotor changes, affect display, and rapport through body language. The Rater Agent evaluated interview skills, MSE formulation, diagnosis, and treatment plan following the STACER checklist. This automated evaluation mechanism generated immediate, detailed feedback aligned with established assessment criteria, bridging the gap between formative learning opportunities and rigorous competency evaluation. With the Rater Agent’s feedback, residents can reflect on what they have learned and seek clarification or confirmation from the Rater Agent to assist with abstract conceptualization before repeating the training session. To validate the Patient Agent, a Psychiatrist Agent was created to automate the interview sessions for evaluation purpose. The Psychiatrist Agent was equipped with DSM-5 diagnostic criteria and the Core of Discipline (COD)-STACER requirements to elicit responses from the Patient Agent. Thus, allowed performance evaluation on the dialogues generated by the Patient Agent. **Zero-shot prompting** was used in all agents. No specific latest learning version was defined in the LLMs. However, performance testing used for this study included GPT-4.1-mini-2025-04-14 for the Patient Agent, GPT-4.1-2025-04-14 for the Rater Agent, GPT-4.1-mini-2025-04-14 for the Psychiatrist Agent, and GPT-5-mini-2025-08-0 used in DeepEval for the performance measure. A complete description of the model and temperature setting for different tasks is provided with the prompts in Section A1.4, Table A1.1.

# A1.1 Patient Agent

The Patient Agent was designed to simulate a psychiatric patient specified in a structured patient profile. It was required to express symptoms verbally and through text-based behaviour, providing realistic behavioural and linguistic manifestations of psychiatric phenomena. The Patient Agent role did not require sophisticated reasoning capabilities but did require rapid response to support real-time interaction. Powered by GPT-4.1-mini with rigorous prompt design for grounding and behavioural regulation, the Patient Agent's function was to provide a simulated clinical assessment environment. GPT-4.1-mini-2025-04-14 was chosen for its fast response time. The Patient Agent was prompted using two prompt files, one to role-play the patient (ie, dos and don’ts, tone, behaviour, and cognitive style), and one to act as described in the case study file without reviewing the symptoms. Please refer to the GitHub repository for the prompt files. The LLM’s tendency to use narrativity as a cognitive resource for sense-making was used to generate a patient’s dialogue for a given profile. The Patient Agent represented a virtual patient based on the chosen patient profile. After a patient profile was selected, the Patient Agent verified the case against the diagnostic criteria for major depressive disorder (MDD) as defined in DSM-5-TR before proceeding. The Patient Agent then conducted a text summarization of the entire profile to provide a two-sentence clinical note as an introduction to the case study at the beginning of the consultation. The consultation session was facilitated through a chat interface. Vocal expression, along with speech-to-text, was created using ElevenLabs’ AI Audio API^38^. ElevenLabs specializes in natural-sounding speech synthesis and generation, making it the choice for creating emotional expression of the human voice. The consultation dialogue between the student and the Patient Agent was stored in the memory for competency assessment.

# A1.2 Rater Agent

The Rater Agent required advanced reasoning capability to provide structured feedback to the students using a COD-STACER assessment form^23^. Combining the case presentation, the dialogue between the student and the Patient Agent, and the patient profile, the Rater Agent generated cross-examination questions and provided the comprehensive assessment. The Rater Agent was powered by GPT-4.1-2025-04-14, a higher reasoning capability model, equipped with psychiatric domain knowledge, supplemented with the diagnostic criteria of the DSM-5 and a detailed rubric, to rate and evaluate the consultation dialogue, case presentation, and answers to cross-examinations. The rubric provided guidelines on rating rapport building, interview technique, interview content, and case presentation. A chat interface was provided for students to ask the Rater agent questions to improve their competency. The Rater Agent returned the assessment on a STACER template by translating narrative interview performance into checklists and detailed feedback items. Additionally, it generated AI-generated Likert scores, guided by the rubric mentioned above, to provide a quick overview of consultation quality. The STACER assessment and detailed feedback enabled residents to reflect on their performance, conceptualize key learning points, and plan for future sessions.

# A1.3 STACER Agentic System Workflow

The SAS workflow was designed around Kolb’s experiential learning cycle, incorporating all four stages. The workflow unfolds across four sequential phases: (1) the resident selects a patient profile to interview; (2) a simulated consultation occurs with the psychiatric Patient Agent (powered by GPT-4.1-mini), mirroring the 55-minute STACER interview; (3) the resident delivers a 15-minute case presentation to the Rater Agent; and (4) the Rater Agent (powered by GPT-4.1) combines the case study file, interview dialogue, presentation and DSM-5 diagnostic criteria to offer feedback. The structured formative feedback was presented in a COD-STACER assessment form. A chat interface was provided for residents to converse with the Rater Agent about the feedback, explore opportunities to extract new knowledge, engage in active reflection, and plan the next session. This design ensures that each training session not only reproduces the authentic rhythm of a clinical encounter but also embeds iterative learning and feedback within the same platform. The workflow was implemented in LangGraph^39^, a Python-based orchestration framework that enables multi-agent coordination and controlled state transitions, allowing the Patient Agent and Rater Agent to interact seamlessly throughout each phase. The LLM’s tendency to use narrativity as a cognitive resource for sense-making was used to generate a patient’s dialogue^40^ for a given profile. Patient’s vocal expression and the resident’s speech-to-text conversion were created using ElevenLabs’ AI Audio API^41^. Please refer to the GitHub repository for the workflow code and the prompt files.

# A1.4 Prompt Usage

This multiple-stage STACER Agentic System contains a Patient Agent and a Rater Agent, at each stage different prompts (Multimedia Appendix 4) were used to instruct the LLMs. Eight system prompts were created for the agents in the SAS. Table 1 provides a summary of the prompts used by the agents in the aforementioned stages of the workflow: (1) patient profile selection, (2) interviewing session, (3) case presentation, and (4) feedback.

Table A1.1**.** Eight prompt files are used in the SAS by various LLM agents in the four workflow stages.

| Stage | Agent | Prompt files | Purpose |
| --- | --- | --- | --- |
| (1) patient profile selection | Patient Agent | Clinical_summary_prompt.txt  model="gpt-4.1-mini"  temperature=0.2 | To describe the patient to the participant with a sentence or two. |
| (2) interviewing session | Patient Agent | virtual_patient_prompt.txt  model="gpt-4.1-mini", temperature=1  Patient_profile_verification_prompt.txt  model="gpt-4.1-mini"  temperature=0 | Define the role of the patient agent and how to behave according to the patient profile. |
|  | Psychiatrist Agent | virtual_student_prompt.txt  model = “gpt-4o-mini”,  temperature=1 | For automated testing where the Psychiatrist Agent is interviewing the patient agent. |
| (3) case presentation | Psychiatrist Agent | student_presentation_prompt.txt  model="gpt-4.1-mini", temperature=0.7 | For automated testing where the Psychiatrist Agent is presenting the case after the interview. |
|  | Rater Agent | evaluator_Q&A_prompt.txt  model="gpt-4.1-mini", temperature=0.7 | Define the role of the Rater agent during the presentation and how to ask follow-up questions to the student. |
| (4) feedback | Rater Agent | virtual_evaluator_prompt.txt  model="gpt-4.1-mini", temperature=0.7  feedback_agent_prompt.txt  STACER_form_prompt.txt  model="gpt-4.1",  temperature=0,  seed = 42 | Define the role of the Rater Agent as a virtual evaluator using STACER form as a formative assessment and additional feedback to the resident. |

# A1.5 Rubric for STACER Psychiatry Clinical Evaluation – Core of Psychiatry

Psychiatry Clinical Evaluation – Core of Psychiatry

This rubric provides a structured framework for evaluating clinical interviewing skills in psychiatry. It is designed for use in teaching, supervision, and assessment of learners such as medical students and psychiatry residents. The framework is organized into five core domains:

Rapport Building

Interview Technique

Interview Content

Case Presentation

Diagnostic Accuracy and Treatment Planning

Each domain is broken into specific expectations drawn directly from the STACER. Every expectation is scored on a 0–N scale, where N reflects the number of criteria in that category. Each criterion includes a brief description and examples of effective and ineffective behaviors.

SECTION 1: INTERVIEW PROCESS

Item: Rapport – Establishes relationship

Score: 0–4

Introduces self

Description: States their name and role clearly at the beginning of the interview.

Good example: “Hello, I’m Dr. Marcus Leighton, one of the psychiatry residents. I’ll be talking with you today to understand what’s been happening.”

Poor example: Walks into the room and immediately asks, “So why are you here?” without introducing self.

Explains interview

Description: Orients the patient to the purpose, structure, and approximate length of the interview.

Good example: “We’ll spend about 30 minutes going over what brought you in, your history, and how things have been recently.”

Poor example: Starts asking diagnostic questions with no explanation of the process.

Respectful

Description: Uses courteous, nonjudgmental language and tone throughout the interaction.

Good example: “Thank you for sharing that, Ms. Alvarez — I know that can be difficult to talk about.”

Poor example: “Why would you do something like that? That doesn’t make sense.”

Open, explorative beginning

Description: Opens with a broad, patient-centered question that encourages narrative expression.

Good example: “Tell me, in your own words, what has been most challenging for you lately.”

Poor example: “Are you here because you’re depressed?”

Item: Rapport – Develops and sustains rapport

Score: 0–3

Remains respectful and nonjudgmental

Description: Maintains a warm, accepting demeanor even during difficult disclosures.

Good example: “I appreciate you trusting me with that, Mr. Chen. Let’s work through it together.”

Poor example: “That’s not a normal reaction — most people wouldn’t respond that way.”

Genuine interest displayed by verbal and non-verbal responses

Description: Shows authentic engagement through attentive posture, affirmations, and reflective statements.

Good example: Leaning forward slightly, nodding, and saying “I hear you — that sounds overwhelming.”

Poor example: Looking at a computer screen or clock while the patient is talking.

Acknowledges patient’s distress with empathic responses

Description: Recognizes and names emotions, validating the patient’s internal experience.

Good example: “It sounds like the last few weeks have felt really frightening for you.”

Poor example: “Okay… anyway, when did this start?”

Item: Control of process – Maintains control of the interview

Score: 0–3

Interrupts politely when required

Description: Redirects excessive or tangential speech gently and respectfully.

Good example: “I’m sorry to interrupt — I want to make sure we cover everything important. May I guide us back to what happened that morning?”

Poor example: “You’re rambling. Just answer the question.”

Redirects when required

Description: Guides the conversation back to clinically relevant areas without invalidating the patient.

Good example: “That sounds important, Ms. Ibrahim, but I want to make sure we come back to the symptoms you noticed last night.”

Poor example: “That’s not what I asked,” said with irritation.

Facilitates organization of disorganized patients

Description: Helps patients structure their thoughts when they struggle to express themselves coherently.

Good example: “Let’s take it step by step — what happened first?”

Poor example: “I can’t follow what you’re saying. Try again.”

Item: Cultural sensitivity – Demonstrates cultural sensitivity

Score: 0–1

Engages patient in a culturally safe manner

Description: Demonstrates awareness of cultural factors, avoids assumptions, and uses inclusive, respectful language.

Good example: “Are there cultural or family beliefs that influence how you’re understanding what’s been happening?”

Poor example: “People from your culture are usually very anxious, so that might explain this.”

Item: Ends the interview – Smoothly closes the interview

Score: 0–2

Attends to timing

Description: Manages the session length appropriately and signals when the interview is nearing its end.

Good example: “We have a few minutes left — is there anything important you’d like to make sure we discuss before we finish?”

Poor example: Suddenly standing up and saying, “Okay, time’s up,” mid-sentence.

Provides a pertinent closing statement

Description: Summarizes key information, explains next steps, and ends with clarity and professionalism.

Good example: “Today we talked about your low mood, sleep changes, and recent stress. Next, we’ll work on making a plan together for support and treatment.”

Poor example: “Alright, I guess that’s it,” with no summary or next steps.

SECTION 2: INTERVIEW TECHNIQUE

Item: Information gathering – Maintains an open, explorative process

Score: 0–3

Non-verbal behavior encourages patient to tell their story

Description: Uses posture, facial expression, and body orientation to promote openness and safety.

Good example: Dr. Rowan Tse sits with an open posture, nodding gently as Ms. Marina Cole shares the onset of her panic symptoms.

Poor example: Dr. Tse leans back with arms crossed, avoiding eye contact as the patient tries to describe her worries.

Listens attentively

Description: Allows the patient to speak without interruption; shows clear presence and concentration.

Good example: Dr. Fiona Porter remains fully focused and silent as Mr. DeShawn Griffith tries to explain his feelings of hopelessness.

Poor example: Interrupting repeatedly with “Uh-huh… but anyway…,” or checking a pager during the patient’s story.

Note taking does not distract from the interview

Description: Uses brief, unobtrusive note taking while maintaining engagement.

Good example: Dr. Lena Gauthier writes short reminders while keeping her eyes mostly on Ms. Alisha Khan, pausing note-taking when emotional content arises.

Poor example: Taking long, continuous notes without looking up, causing the patient to fall silent because they feel ignored.

Item: Information gathering – Uses a facilitative questioning style

Score: 0–6

Questioning follows a logical but flexible sequence

Description: Moves through topics in a coherent order while allowing deviation when clinically appropriate.

Good example: “You mentioned the anxiety began after the move — can you tell me about what that transition was like?”

Poor example: Jumping from sleep to childhood to hallucinations with no connection or flow.

Asks clear questions in plain language

Description: Avoids jargon; uses understandable wording.

Good example: “When you say you felt ‘off,’ what exactly did that feel like?”

Poor example: “Were there any prodromal features preceding the affective dysregulation?”

Avoids leading questions

Description: Questions do not suggest a preferred answer.

Good example: “What thoughts were going through your mind when that happened?”

Poor example: “You weren’t actually serious about harming yourself, right?”

Avoids stacked (multiple) questions

Description: Limits questions to one idea at a time.

Good example: “How has your appetite been?”

Poor example: “How’s your sleep, appetite, energy, and concentration?”

Moves effectively between open and closed questions

Description: Uses open questions to explore and closed questions to clarify.

Good example: “What was that week like for you?” followed later by “And how many hours were you sleeping on average?”

Poor example: Using only yes/no questions even when elaboration is needed.

Facilitates expression of emotions

Description: Encourages the patient to reflect on and articulate feelings.

Good example: “When your partner said that to you, what emotions came up for you?”

Poor example: Switching immediately to fact-finding whenever emotion appears.

Item: Information gathering – Pursues important information

Score: 0–3

Appropriately responds to informational and affective cues

Description: Notices and explores emotional and content-based openings.

Good example: The patient tears up, and Dr. Kaitlyn Mercer says, “I can see this is painful — what’s coming up for you right now?”

Poor example: Ignoring tears and continuing with, “Have you ever had surgery?”

Pursues symptom detail

Description: Asks the necessary follow-up questions to clarify severity, duration, pattern, and associated factors.

Good example: “You said the voices come in the evening — how long do they last? Do they command you to do anything?”

Poor example: Accepting vague statements without clarification.

Asks for clarification

Description: Ensures understanding by checking ambiguous statements.

Good example: “When you say you ‘checked out,’ do you mean you felt numb or that you lost track of time?”

Poor example: Assumes meaning and moves on without verifying.

Item: Interview technique – Maintains Flow

Score: 0–5

Supportively confronts inconsistencies

Description: Identifies contradictions gently and explores them collaboratively.

Good example: “Earlier you mentioned you hadn’t been sleeping, but just now you said you sleep fine. Help me understand how both feel true for you.”

Poor example: “That doesn’t make sense — which one is it?”

Appropriately deals with unusual, difficult, or distressing content

Description: Remains calm, steady, and grounded; does not show shock, judgment, or avoidance.

Good example: When Mr. Eli Njoroge describes his intrusive violent thoughts, Dr. Priya Raman maintains a steady tone and thanks him for his honesty.

Poor example: Appearing visibly alarmed or rushing to change the subject.

Comfortably allows silence to facilitate further expression

Description: Uses intentional pauses to encourage elaboration.

Good example: After Ms. Tara Leduc mentions a traumatic event, Dr. Arjun Singh stays quiet for a few seconds, giving her space to continue.

Poor example: Immediately filling every silence with another question.

Reframes when required

Description: Offers alternative perspectives that clarify or contextualize the patient’s experience.

Good example: “It sounds like you see yourself as a burden, but from what you’ve said, your family seems very invested in your wellbeing.”

Poor example: “You’re just looking at it wrong.”

Summarizes when appropriate

Description: Periodically synthesizes what has been said to ensure shared understanding.

Good example: “So far I’m hearing that your anxiety increased after the layoff, and you’ve been struggling with sleep and appetite. Do I have that right?”

Poor example: Never checking understanding; or giving long, monologue-like summaries that interrupt the patient’s flow.

SECTION 3: INTERVIEW CONTENT

Item: Elicits a complete, relevant, and accurate history – Identifies the person

Score: 0–1

Obtains complete demographic information

Description: Collects essential identifying information such as age, gender identity, living situation, occupation, and relationship status.

Good example: “So before we continue, can I confirm a few details? You’re 32, living with your partner in Oakridge, and you work as a line cook — is that right?”

Poor example: Does not ask any demographic details and later guesses age or circumstances, leading to inaccurate charting.

Item: Elicits a complete, relevant, and accurate history – Identifies the presenting complaint(s) or problem(s) and its/their history

Score: 0–5

Obtains data on presenting complaint(s) or problems

Description: Clarifies what brought the patient in, capturing symptoms in the patient’s own words.

Good example: “What made you decide to come in today, Mr. Vargas?” followed by detailed exploration.

Poor example: Immediately labeling the issue without asking what the patient is experiencing.

Assesses premorbid state

Description: Establishes baseline functioning prior to symptom onset.

Good example: “Before these panic episodes began, how were you doing day-to-day? What was your usual energy and mood like?”

Poor example: Assuming the patient has always been functioning poorly.

Assesses stressors related to presenting illness

Description: Identifies contributing psychosocial or environmental stressors.

Good example: “You mentioned the symptoms started after the breakup. What was happening in your life around that time?”

Poor example: Overlooking major stressors the patient previously mentioned.

Assesses previous illness episodes and compares them with the current episode

Description: Explores pattern, recurrence, severity, and past similarities.

Good example: “You said you experienced something similar five years ago. How does this episode compare in terms of intensity?”

Poor example: Ignoring the patient’s history of similar episodes.

Identifies treatment interventions and response for this episode

Description: Clarifies what has been tried, including medication, therapy, or coping strategies, and whether they helped.

Good example: “You tried sertraline for two weeks — what changes did you notice while on it?”

Poor example: “You’re on meds, right?” without exploring adherence or response.

Item: Elicits a complete, relevant, and accurate history – Screens for symptoms relevant to the differential diagnosis

Score: 0–4

Reviews primary criteria of other relevant diagnoses

Description: Screens for symptoms related to mood disorders, anxiety disorders, psychosis, mania, PTSD, etc.

Good example: “Have there been times when you felt unusually energized or needed little sleep?”

Poor example: Only asking, “You’re not psychotic, right?”

Reviews substance use and abuse

Description: Explores use of alcohol, cannabis, stimulants, opioids, and other substances.

Good example: “You mentioned drinking more lately — how often are you drinking, and how much each time?”

Poor example: “You don’t use drugs, do you?” said in a judgmental tone.

Assesses impact of substance use on person and others

Description: Looks at consequences such as occupational, relational, or legal issues.

Good example: “How has your drinking affected your relationships or work performance?”

Poor example: Only asking about quantity but not consequences.

Assesses motivation to change substance use (if appropriate)

Description: Explores ambivalence, readiness, and goals.

Good example: “Where do you feel you are right now in terms of wanting to cut down or make changes?”

Poor example: “You should really stop using,” delivered without assessment or collaboration.

Item: Elicits a complete, relevant, and accurate history – Ensures safety

Score: 0–5

Completes an appropriate risk assessment (self-harm, aggression, self-care, competency)

Description: Screens for suicidal ideation, intent, plan, access to means, homicidal thoughts, ability to perform ADLs, and decision-making capacity.

Good example: “When you said you felt like giving up, did you mean having thoughts of ending your life?”

Poor example: “You’re not suicidal, right?” asked in a dismissive or rushed manner.

Reviews current medication(s), dosage(s), and response

Description: Determines what medications are prescribed, whether the patient takes them, and how effective they are.

Good example: “You’re taking quetiapine 100 mg at night. Have you noticed changes in sleep or mood since starting it?”

Poor example: Forgetting to ask about adherence or dosage.

Reviews use of over-the-counter products

Description: Checks for supplements, herbal products, and non-prescription medications.

Good example: “Do you use anything like melatonin, vitamins, or herbal remedies?”

Poor example: Ignoring OTC substances that may interact with prescriptions.

Elicits complete history of side effects

Description: Explores unwanted symptoms related to current medications.

Good example: “Since starting your antidepressant, have you noticed nausea, headaches, restlessness, or any sexual side effects?”

Poor example: Never asking about side effects.

Defines allergy status

Description: Clarifies medication allergies, reactions, and severity.

Good example: “You said you had a reaction to amoxicillin — what happened when you took it?”

Poor example: “You don’t have any allergies, right?” without exploration.

Item: Elicits a complete, relevant, and accurate history – Identifies relevant past history

Score: 0–4

Reviews past medical history including family history of medical disorders

Description: Explores chronic illnesses, surgeries, conditions, and family medical issues.

Good example: “Any history of thyroid issues, seizures, or other long-term conditions?”

Poor example: Skipping medical history altogether.

Reviews past psychiatric history

Description: Previous diagnoses, hospitalizations, treatments, and therapy experiences.

Good example: “Have you ever been hospitalized for your mental health before?”

Poor example: Asking, “You’ve never seen a psychiatrist, right?” assuming no history.

Reviews family psychiatric history

Description: Screens for mental illness among biological relatives.

Good example: “Has anyone in your family struggled with mood disorders, psychosis, or substance use?”

Poor example: Not asking about family history despite strong genetic indicators.

Reviews forensic history

Description: Screens for legal issues, charges, or incarceration.

Good example: “Have you had any legal difficulties or charges in the past?”

Poor example: Avoiding the topic due to discomfort.

Item: Elicits a complete, relevant, and accurate history – Identifies the developmental and psychosocial history

Score: 0–12

Family history and dynamics

Description: Explores household environment, relationships, roles, and conflicts.

Good example: “Who lived in your home growing up, and what were those relationships like?”

Poor example: Only asking, “Were your parents together?” with no further exploration.

Gestational and perinatal history

Description: For pediatric or relevant adult cases, explores pregnancy complications, birth complications, and early health.

Good example: “Do you know if there were any complications during your mother’s pregnancy or your birth?”

Poor example: Never considering perinatal history even in neurodevelopmental assessments.

Childhood and adolescent development

Description: Reviews developmental milestones, social functioning, academic performance, and behavioral issues.

Good example: “How were things for you in school growing up?”

Poor example: Skipping childhood entirely.

Occupational history and current functioning

Description: Explores employment history, performance, satisfaction, and functional impairment.

Good example: “How has your concentration at work changed since symptoms began?”

Poor example: Asking only, “Do you have a job?” with no follow-up.

Relationship history

Description: Explores past and current intimate relationships, patterns, and significant experiences.

Good example: “How have your relationships been affected by your mood changes?”

Poor example: Avoiding questions about relationships due to discomfort.

Past and current history of abuse

Description: Screens compassionately for emotional, physical, sexual, or financial abuse.

Good example: “Sometimes people who feel this way have gone through difficult or harmful experiences. Has anything like that happened to you?”

Poor example: “You haven’t been abused, have you?” asked dismissively.

Current supports

Description: Identifies support networks such as family, friends, community, or services.

Good example: “Who do you feel most comfortable turning to when things get difficult?”

Poor example: Assuming the patient has no supports.

Relevant cultural identities, migration history, and trauma/stressors

Description: Explores cultural background, migration experiences, discrimination, or cultural stress.

Good example: “Has your experience moving to Canada influenced the way you’re feeling now?”

Poor example: Making assumptions based on ethnicity.

Spirituality

Description: Assesses spiritual or religious beliefs relevant to coping or understanding illness.

Good example: “Are spiritual or religious beliefs important in how you make sense of what you’ve been experiencing?”

Poor example: “Religion doesn’t matter here,” said dismissively.

Identifies social and cultural supports including family, kin networks, and communities

Description: Looks at community involvement, extended family, and cultural groups.

Good example: “Are there community groups or extended family members who play an important role in your life?”

Poor example: Overlooking involvement in cultural communities.

Identifies social and cultural stressors and systemic inequities

Description: Explores experiences with discrimination, poverty, unsafe conditions, or systemic barriers.

Good example: “Have financial strain or workplace discrimination played a role in your stress recently?”

Poor example: Ignoring systemic issues even when the patient mentions them.

Explores patient’s explanatory model of illness

Description: Asks how the patient understands their symptoms and what they believe is causing their distress.

Good example: “What do you think is happening when these thoughts come on?”

Poor example: Assuming the patient’s interpretation without asking.

Item: Conducts a formal Mental State Examination as indicated

Score: 0–7

Appropriately adapts the Mental Status Examination to be culturally competent

Description: Interprets behavior, affect, and cognition within the patient’s cultural context; avoids mislabeling normal culturally mediated behaviors as psychopathology.

Good example: Dr. Helena Brooks asks, “In your cultural or spiritual tradition, is speaking with ancestors considered a normal experience?” before concluding whether the patient’s experiences are hallucinations.

Poor example: Immediately labeling culturally normative spiritual practices as “psychosis” without clarification.

Assesses mood symptoms

Description: Evaluates subjective mood (“how the patient feels”) and observed affect.

Good example: “How would you describe your mood lately — sad, anxious, flat, overwhelmed?”

Poor example: Only stating “mood normal” without asking or observing anything.

Assesses anxiety symptoms

Description: Screens for worry, physiological arousal, panic symptoms, and avoidance behaviors.

Good example: “Do you ever experience sudden episodes of intense fear, racing heart, or shortness of breath?”

Poor example: Never exploring anxiety even when the patient clearly describes worry.

Assesses psychotic symptoms

Description: Screens for hallucinations, delusions, disorganized thought, or abnormal perceptions.

Good example: “Have you had any experiences where you heard or saw things that others cannot?”

Poor example: Asking, “You’re not hallucinating, right?” in a dismissive tone.

Assesses insight and judgment

Description: Evaluates how the patient understands their condition and how they make decisions.

Good example: “When these thoughts come up, how do you usually respond?”

Poor example: Writing “insight poor” without asking any relevant questions.

Assesses cognition if relevant

Description: Explores orientation, attention, memory, and executive function.

Good example: “Just to check focus — can you spell the word WORLD backwards?”

Poor example: Skipping cognition despite the patient describing memory issues.

Considers intellectual function if relevant

Description: Uses history, education level, language, and interaction to estimate global intellectual functioning.

Good example: Asking, “How was school for you growing up? Did you have any learning difficulties?” when relevant.

Poor example: Assuming low intelligence because of socioeconomic status or manner of speech.

SECTION 4: CASE PRESENTATION

Item: Defines limitations of the data – Identifies issues in the information gathering process

Score: 0–3

Reports on the reliability of the patient (with examples)

Description: Assesses whether the patient’s information is consistent, coherent, and trustworthy.

Good example: “Information about substance use may be unreliable, as Mr. Dalton gave conflicting accounts regarding his drinking.”

Poor example: Ignoring obvious inconsistencies without commenting.

Reports on the accessibility of the patient (with examples)

Description: Notes if guardedness, disorganization, or distress interfered with data collection.

Good example: “Ms. Everett was tearful and withdrawn during questions about trauma, limiting detail.”

Poor example: Pretending the patient was fully cooperative when major barriers existed.

Identifies deficits in the interview and their potential effect on the data collection

Description: Acknowledges if time limits, missed questions, or incomplete exploration impacted accuracy.

Good example: “Due to time constraints, sleep history was only partially explored, which may affect diagnostic clarity.”

Poor example: Presenting incomplete data as if it were comprehensive.

Item: Presentation skills – Provides a coherent, accurate summary of the case

Score: 0–6

Uses descriptive terms correctly (e.g., delusions)

Description: Applies psychiatric terminology appropriately and accurately.

Good example: “He describes a fixed, false belief that neighbors are monitoring him, consistent with persecutory delusions.”

Poor example: Calling ordinary worries “delusions.”

Presents case in an orderly, concise, systematic manner that is sufficiently detailed

Description: Provides a structured, logical narrative that includes all relevant domains.

Good example: Following a clear structure: chief complaint → history → MSE → risk → formulation.

Poor example: Jumping back and forth between timelines with no structure.

Accurately reports the Mental State Examination

Description: Summarizes key MSE findings with clarity.

Good example: “Affect was constricted; speech was slowed; thought process was goal-directed.”

Poor example: “MSE normal,” without details.

Accurately reports the risk assessment (self-harm, aggression, self-care, competency)

Description: Presents risk findings clearly.

Good example: “He reports passive suicidal ideation with no plan or intent.”

Poor example: “No risk,” even though ideation was disclosed.

Accurately reports the Mental Status Examination

Description: Same function as above; preserved for fidelity to STACER form.

Good example: “Insight was limited; judgment impaired during episodes of intoxication.”

Poor example: Contradictory or missing MSE details.

Identifies relevant comorbidities

Description: Notes concurrent conditions that affect diagnosis or treatment.

Good example: “Generalized anxiety symptoms significantly worsen depressive episodes.”

Poor example: Ignoring comorbid panic symptoms when describing mood disorder.

Item: Synthesizing skills – Diagnostic thinking

Score: 0–6

Presentation emphasizes necessary information to support and defend diagnosis and differential

Description: Provides evidence-based reasoning for diagnostic decisions.

Good example: “Low mood, anhedonia, sleep disruption, and guilt for more than two weeks support MDD; lack of elevated mood or decreased need for sleep makes bipolar less likely.”

Poor example: “Probably depression,” without evidence.

Provides a working diagnosis including comorbidities supported by evidence from the interview

Description: Gives a primary diagnosis and any comorbid conditions with justification.

Good example: “Working diagnosis: Major Depressive Disorder with comorbid Social Anxiety Disorder.”

Poor example: Listing diagnoses not discussed in interview.

Provides a differential diagnosis supported by evidence

Description: Identifies reasonable alternatives with rationale.

Good example: “Consider GAD, given pervasive worry; rule out PTSD due to trauma history.”

Poor example: “Could be anything.”

Discusses comorbidities and interplay between diagnoses

Description: Describes how conditions interact or complicate each other.

Good example: “Her alcohol use worsens depressive symptoms and impairs sleep, contributing to anxiety.”

Poor example: Mentioning comorbidities without integration.

Provides a realistic prognosis

Description: Describes likely course based on history, severity, and treatment response.

Good example: “Given the chronicity and prior good response to SSRIs, prognosis with treatment is fair-to-good.”

Poor example: “You should be fine,” without justification.

Describes barriers to compliance or optimal treatment

Description: Notes factors like stigma, finances, cognition, transportation, or mistrust.

Good example: “Limited transportation may affect therapy attendance.”

Poor example: Blaming the patient: “He doesn’t care enough to follow treatment.”

Item: Synthesizing skills – Formulation

Score: 0–6

Identifies biological factors

Description: Notes genetics, medical comorbidities, substance use, neurobiology.

Good example: “Family history of bipolar disorder increases vulnerability.”

Poor example: Ignoring known medical contributors.

Identifies psychological factors

Description: Explores coping styles, trauma, personality traits.

Good example: “Perfectionistic tendencies amplify stress.”

Poor example: “It’s all in their head.”

Identifies social factors

Description: Notes relationships, supports, stressors, socioeconomic context.

Good example: “Job loss preceded symptom escalation.”

Poor example: Overlooking obvious environmental triggers.

Identifies cultural factors

Description: Incorporates cultural identity, migration, and explanatory models.

Good example: “Stigma in her cultural community contributes to delayed help-seeking.”

Poor example: Assuming culture is irrelevant.

Provides an integrated account of interplay between biopsychosocial components

Description: Synthesizes biological, psychological, social, and cultural factors into a coherent narrative.

Good example: “Biological vulnerability, perfectionistic thinking, and lack of support interact to produce and maintain her depressive episode.”

Poor example: Listing factors in isolation without explaining how they fit together.

Identifies prominent internal conflicts and/or cognitive distortions that influence the patient’s presentation

Description: Explicitly describes key internal conflicts (e.g., autonomy vs. responsibility) and/or distorted beliefs (e.g., all-or-nothing, catastrophizing) that shape symptoms and behavior.

Good example: “She holds a rigid belief that ‘a good mother never struggles,’ creating an internal conflict between her real limitations and an unrealistic ideal, which fuels guilt and hopelessness.”

Poor example: “She feels bad about herself,” without identifying any underlying conflicts or distorted thoughts.

SECTION 5: TREATMENT PLAN

Item: Identifies information required to consolidate the diagnosis

Score: 0–1

Identifies further, appropriate, and cost-effective biopsychosocial investigations required to confirm the diagnosis or provide optimal care to the patient

Description: Selects ONLY the investigations needed to meet or rule out DSM-5 criteria (core symptoms, duration, impairment, exclusion conditions). Recommendations must be clinically relevant and cost-effective (e.g., targeted labs, collateral, records review).

Good example: “To confirm DSM-5 Major Depressive Disorder and rule out exclusionary medical conditions, Dr. Mara Singh orders TSH and B12, and requests collateral from the patient’s partner regarding functional impairment.”

Poor example: Ordering broad, unnecessary tests: “Let’s get an MRI, CT scan, autoimmune panel, and toxicology panel to confirm your mild anxiety.”

Item: Communicates a comprehensive treatment plan

Score: 0–8

Utilizing a biopsychosocial matrix defines an immediate, short-term, and long-term treatment plan

Description: Creates a structured plan addressing biological, psychological, and social domains that aligns with DSM-5 diagnosis and CANMAT treatment sequencing.

Good example: “Immediate safety planning for suicidal ideation; short-term: start first-line SSRI; long-term: reintroduce CBT and vocational support per CANMAT guidance for moderate MDD.”

Poor example: “We’ll just check in next month,” with no short- or long-term planning.

Recommends specific biological therapies (pharmacotherapy, ECT, TMS, etc.) for the patient

Description: Chooses biological interventions based on CANMAT first-, second-, or third-line guidelines, tailored to the DSM-5 diagnosis and severity.

Good example: “For Mr. Leo Santiago’s severe DSM-5 depression, recommend a first-line SSRI; if no improvement after adequate trial, CANMAT suggests augmentation or ECT.”

Poor example: “Start benzodiazepines for depression,” or recommending TMS as first-line for mild symptoms.

Recommends a specific psychotherapeutic approach for the patient

Description: Selects psychotherapy appropriate for the DSM-5 diagnosis and supported by CANMAT evidence (e.g., CBT, IPT, exposure therapy).

Good example: “Ms. Aisha Romero’s DSM-5 panic disorder aligns with CANMAT first-line CBT with exposure.”

Poor example: “Any therapy is fine,” with no rationale.

Considers social and cultural factors in all aspects of treatment planning

Description: Integrates cultural identity, language, explanatory models, stigma, systemic barriers, and social supports into care.

Good example: “Because Mr. Tariq El-Sayed prefers therapy in Arabic and medication is stigmatized in his community, prioritize CANMAT-supported CBT and connect him with a culturally matched therapist.”

Poor example: “Culture doesn’t affect treatment,” or ignoring stated cultural needs.

Identifies appropriate collaborations with family, community, or other service providers

Description: Coordinates care with family physicians, social workers, addictions services, shelters, housing supports, and culturally relevant organizations when indicated.

Good example: “Dr. Harlow contacts the patient’s family doctor to review medication history and connects her with a housing worker to stabilize living conditions.”

Poor example: “I don’t collaborate with outside providers — the patient can tell them on their own.”

Provides evidence for the efficacy of the treatment plan

Description: Explains why chosen treatments are appropriate based on CANMAT evidence tiers and known treatment effects for DSM-5 conditions.

Good example: “CBT is a CANMAT first-line treatment for GAD, with strong evidence for reducing worry severity within 6–12 sessions.”

Poor example: “This medication works for everyone,” or offering no rationale.

Identifies the expected benefits and risks of the treatment plan

Description: Describes benefits, therapeutic timelines, adverse effects, and risk considerations appropriate to the chosen CANMAT-supported interventions.

Good example: “Sertraline may improve mood within 2–4 weeks; possible side effects include nausea and headache.”

Poor example: “There are no side effects — just take it.”

Identifies the follow-up procedure

Description: Outlines follow-up timing, monitoring needs, and safety checks consistent with CANMAT guidelines (e.g., 2–4 week follow-up after medication initiation).

Good example: “We’ll meet again in two weeks to review response and side effects; if no improvement by 6–8 weeks, we’ll adjust based on CANMAT guidance.”

Poor example: “Follow up whenever you want.”
