# Supplementary material for: Enhancing Psychiatry Training Using an Agentic AI Simulated Consultation Tool: Prospective Cohort Study
Source: JMIR Med Educ. 2026 Jul 21;12:e88580. doi: 10.2196/88580 (PMC13387278; doi:10.2196/88580)
Supplement: Multimedia Appendix 3 [file mededu-v12-e88580-s003.docx]

Appendix 3: Case Study Examples

# A3.1 DSM-5-TR Real Patient Profile Example

Each case study was described by five sections of information, including:

**Section 1: Case Study**

The case study summarizes the information from the real case described in the DSM-5. Provides information about who the patient is, why a psychiatrist is seeing them, and how their symptoms are presented

**Section 2: Behaviour**

The bullet points describing the patient’s behaviour during the interview. Used to instruct the LLM how to act and how to answer questions (eg, ‘answers questions in short sentences’, ‘makes no eye contact’, ‘cooperative’, etc.)

**Section 3: Depressive Symptoms for DSM-5**

Listing the MDD symptoms with brief descriptions that the patient presents with. Informs the LLM of which symptoms it must exhibit.

**Section 4: Patient Profile**

The patient profile section provides the basic information about the patient (demographic, medical history, etc.) used to help the LLM answer questions about the patient’s medical and personal history.

**Section 5: Diagnosis**

This section provides a full diagnosis of the patient taken from the original case study. This is used to inform the user at the end what the correct diagnosis would be.

# A3.2 Synthetic Case Study

Using ChatGPT-5, we generated patient demographic profiles, and Python scripts were used to link these profiles to relevant symptom presentations, allowing us to systematically evaluate Patient Agent performance. Using DSM-5-TR diagnostic criteria for major depressive disorder (MDD), we generated 227 synthetic case studies capturing all valid symptom combinations meeting the diagnostic threshold (≥5 symptoms, including depressed mood or anhedonia). Although prior work only identified 119 combinations [1], our expanded set enabled comprehensive testing of model behaviour across the full diagnostic space. Each case included demographic data, behavioural descriptors, symptom profiles, and medical history.

Following the case studies from the DSM-5-TR, each of the 227 case studies was enriched with a complete narrative below:

- Presenting complaint (one sentence expression).
- History of present illness (timeline of symptoms, context, functional impairment).
- Psychiatric history, family history, medical history, and substance use.
- Mental status exam summary.
- Symptom list (mapped to DSM-5-TR).
- Diagnosis

[1] Park SC, Kim JM, Jun TY, Lee MS, Kim JB, Yim HW, Park YC. How many different symptom combinations fulfil the diagnostic criteria for major depressive disorder? Results from the CRESCEND study. Nord J Psychiatry Taylor and Francis Ltd; 2017 Apr 3;71(3):217–222. PMID:27981876


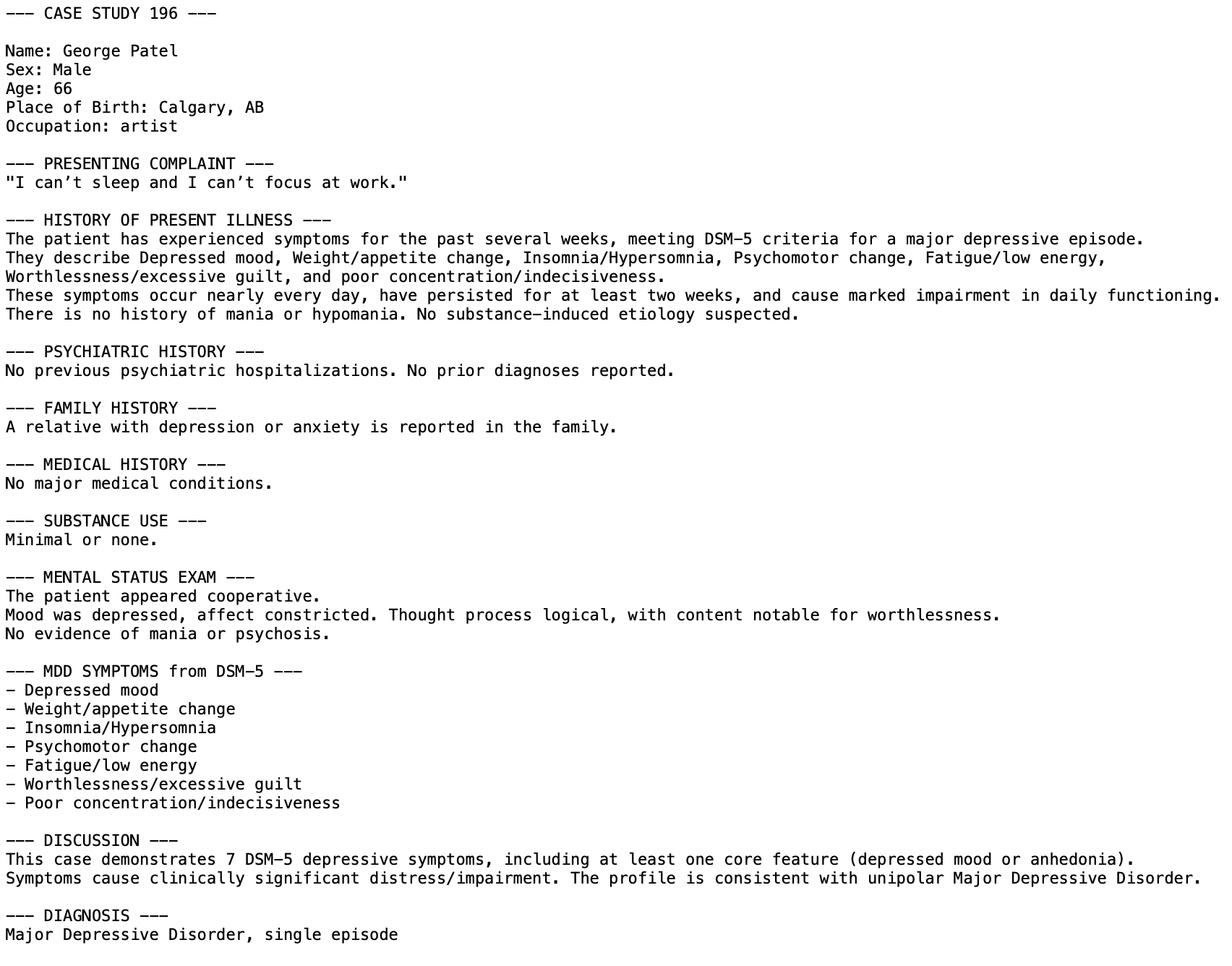


Figure A3.1 Synthetic patient profile generated by ChatGPT-5 and Python script.
