# Supplementary material for: Enhancing Psychiatry Training Using an Agentic AI Simulated Consultation Tool: Prospective Cohort Study
Source: JMIR Med Educ. 2026 Jul 21;12:e88580. doi: 10.2196/88580 (PMC13387278; doi:10.2196/88580)
Supplement: Multimedia Appendix 4 [file mededu-v12-e88580-s004.docx]

Appendix 4: Detailed Description of the Performance Measure

The SAS performance measure was designed to evaluate the Patient Agent and the Rater Agent. Based on the STACER assessment framework (Appendix B), a synthetic benchmark dataset was created to evaluate the Patient Agent's performance in simulating depressive symptoms. The performance of the SAS was evaluated based on 1) the Patient Agent’s imitation of a patient with MDD; 2) time complexity for the Patient Agent and Rater Agent; 3) the learner feedback on their experience with the system; and 4) professional ratings on the feedback and ratings provided by the Rater Agent.

1. To evaluate the linguistic and technical performance of the Patient Agent, eight evaluation metrics from ConfidenceAI’s DeepEval (v.3.4.8) were measured using the GPT-5-mini model for its speed and suitability for domain specific tasks. Instead of measuring performance on turn-by-turn utterances, we measured the performance at the conversational level. A Virtual Psychiatrist external to the system was created to elicit multiturn responses from the Patient Agent. This automated testing on a synthetic benchmark dataset was conducted prior to the feasibility test. Additional elaborate performance measures were required to analyze the multiturn conversational flow. We examined eight key metrics in the Patient Agent: accuracy in simulating the patient profile, clarity in the patient’s expression, professional tonality, medical faithfulness, bias, toxicity, turn relevance and role adherence. The definitions of these metrics are provided in the Multimedia Appendix B**.**
2. Five non-psychiatrist clinical research team members with backgrounds in psychiatry/psychology within our group, along with 9 psychiatry residents, were recruited to act as students to evaluate the Patient Agents. Each person provided Likert-like quantitative feedback, rated on a scale from 0 (very poorly) to 5 (excellent) on each item.
3. The participating psychiatrists within the team assessed the grading and feedback from the Rater Agent (scores ranging from 1 to 5), along with the cross-examination questions presented to the student. We compared the Rater Agent’s assessment with that of the practicing psychiatrists. The psychiatrist provided independent STACER feedback and quality scores on the simulation runs. The two-way interclass correlation coefficient (ICC) agreement test using R’s (version 4.5.1) Psych package was used to measure inter-rater reliability between the Rater agent and the psychiatrist. Based on the 95% confidence interval of the ICC estimate, values less than 0.5, between 0.5 and 0.75, between 0.75 and 0.9, and greater than 0.90 indicate poor, moderate, good, and excellent reliability, respectively.
4. Simulation results from the participants were manually analyzed and compared with the behaviour and symptoms.

# A4.1 DeepEval Performance Evaluation Metrics

This section provides the definitions of the eight DeepEval performance evaluation metrics used for automated testing.

| Term | Definition |
| --- | --- |
| Correctness | Correctness compares the patient’s concatenated utterances against the patient profile with five scoring ranges (0-2), (3-5), (6-7), (8-9), (10-10) representing "Factually incorrect", "Mostly incorrect", "Mostly correct", "Correct but missing minor details", “Factually correct", respectively. |
| Clarity | Clarity measures the patient’s expression as straightforward and easy to understand, without confusion or vagueness. |
| Professionalism | Professional tonality measures whether the patient demonstrates expertise, chooses a professional tone, and shows respectfulness. |
| Medical Faithfulness | Medical faithfulness of the symptom presentation measures the degree symptoms expressed by the patient match the description in the patient profile. |
| Bias | Bias (a DeepEval's safety metric) measures whether the patient exhibits gender, racial, or political bias. |
| Toxicity [1] | Toxicity (a DeepEval's safety metric) measures harmful content, including toxicity, identity attack, insult, profanity, and threat. |
| Turn Relevancy | Turn relevance (a multiturn conversation [2] metric) considers the relevance between two adjacent utterances across the entire discussion. |

[1] Luong TS, Le T-T, Van LN, Nguyen TH. Realistic evaluation of toxicity in large language models. 2024 May 20; Available from: http://arxiv.org/abs/2405.10659

[2] Li Y, Shen X, Yao X, Ding X, Miao Y, Krishnan R, Padman R. Beyond single-turn: a survey on multi-turn interactions with large language models. 2025 May 14; Available from: http://arxiv.org/abs/2504.04717

# A4.2 Time-Complexity

We have also measured time complexity to study the response time of the Patient Agent and Rater Agent to determine if the SAS can provide a real-time experience. The time complexity measure included the average duration of each conversation turn between the Virtual Psychiatry and the Patient Agent, as well as the average time required for the Rater Agent to provide feedback and grading.

The results for the time complexity are provided in **Table A2.1**. The table summarizes the time complexity of agent reasoning time, including the average number of turns required to complete the interview, the average duration of each turn, and the Rater Agent's reasoning time to provide feedback.

Table A2.1**.** Time complexity measures.

| Measurements | Mean (SD) |
| --- | --- |
| Average number of turns per interview | 25.06 (3.21) |
| Average duration per turn length (seconds). | 2.86 (0.46) |
| Average RATER processing time per session (minutes) | 3.43 (0.54) |

# A4.3 Feedback and Evaluation

The LABS feedback and evaluation allow the residents to converse with the Rater Agent (Evaluator), obtain a copy of the STACER assessment report, and receive detailed feedback. Following the Core STACER Assessment Form from the University of Toronto^^[[1]](#footnote-1)^^, the SAS uses the STACER format as one of its formats. A copy of the STACER report generated by the SAS can be found [here](https://drive.google.com/drive/folders/1bY__iyzbmtPudjhyAqIA6cXcJFXuM6EB)^^[[2]](#footnote-2)^^.

1. Official Unversity of Toronto STACER Form: <https://psychiatry.utoronto.ca/sites/default/files/assets/files/core-stacer-assessment-formupdateddecember2022-v2_0.pdf> [↑](#footnote-ref-1)
2. SAS generated STACER Form: [Sample STACER FORM.pdf](https://drive.google.com/file/d/1zYwL3gTIfAyRN5creZqOl5V3x1ADWxPH/view?usp=drive_link) [↑](#footnote-ref-2)
