# Supplementary material for: Enhancing Psychiatry Training Using an Agentic AI Simulated Consultation Tool: Prospective Cohort Study
Source: JMIR Med Educ. 2026 Jul 21;12:e88580. doi: 10.2196/88580 (PMC13387278; doi:10.2196/88580)
Supplement: Checklist 1 [file mededu-v12-e88580-s005.pdf]

**Development, Evaluation, and Assessment of Large Language Models (*DEAL*) Checklist Part B**						
**Section / Topic**	**No.**	**Item**	**Response**	**Page Number**	**No**	**N/A**
**Model Information & Study Setup**	1.1	LLM Model Information	Provide name(s), version(s), and dates of use. Indicate if using default or modified version(s).	Pg.11 Appendix 1		
	1.2	Model Selection & Usage Purpose	Briefly explain the rationale for choosing the specific LLM and its intended application (e.g., content generation, classification).	Appendix 1		
	1.3	Parameter Adjustments*	List basic parameter adjustments (e.g., temperature, max tokens, penalties). State if using default settings.	Appendix 1		
	1.4	API/Tool Information*	Specify the API or platform (e.g., OpenAI API, Hugging Face) used to interact with the LLM.	Pg. 9, 11		
	1.5	Custom Instructions	Describe any specific instructions or system prompts given to the model (e.g., "You are a helpful assistant...").	Appendix 5		
	1.6	Most Recent Training Date	Provide the most recent training date of the LLM model used.	Appendix 1		
	1.7	Annotation Process	Outline any annotation processes, including team roles, guidelines, and quality control measures.			N/A
	1.8	Compute Resources*	Specify compute resources (e.g., GPU/CPU hours, cost) used in model usage or fine-tuning.	Pg. 14 Appendix 4 (Table A2.1)		
	1.9	Ethical Considerations	Indicate Institutional Review Board (IRB) or ethics committee approvals, and patient consent/waivers, as applicable.			N/A
	1.1	Funding and Disclosures	Disclose funding sources and any potential conflicts of interest (COI).	Pg. 28-29		
**Prompt Engineering**	2.1	Prompting Strategies	Describe the key prompts tested or used, including techniques such as zero-shot, few-shot, or chain-of-thought (CoT) prompting.	Appendix 1		

	2.2	Iterative Prompting	Describe any iterative process used to refine prompts based on model outputs.			N/A
	2.3	Example Prompts	Provide examples of the prompts that were most effective. Optionally, include examples as supplemental material.	Appendix 5		
	2.4	Multi-Step Prompting*	If multiple stages of prompts were used (e.g., multi-agent scenarios), describe the sequence and rationale.	Appendices 1 and 5		
**Output Evaluation**	3.1	Basic Evaluation Methods	Describe how outputs were evaluated (e.g., human evaluation, automated quality checks).	Pg. 13-14 Appendix 4		
	3.2	Metrics for Evaluation	List any metrics used to assess the outputs (e.g., coherence, fluency, relevance). If no formal metrics, describe qualitative observations.	Pg. 13-14 Appendix 4		
	3.3	Post-Processing of Outputs*	Indicate if any post-processing (e.g., filtering, cleaning, or human review) was applied to improve the final output.	Appendix 4		
**Bias and Fairness Evaluation**	4.1	Basic Bias Detection	Provide general approach to identifying bias, including qualitative observations of fairness issues in model outputs.	Pg. 13		
	4.2	Synthetic Data Bias*	For synthetic data, describe basic checks to ensure fairness and data quality.			N/A
	4.3	Fairness Observations	Note subjective observations of bias, including cases where model may exhibit consistent bias in responses.	Pg. 13-14		
**Model Stochasticity Management**	5.1	Basic Consistency Check	Describe steps taken for multiple queries to ensure consistency, including general observations of response variability.	Pg. 13-14, 17-19 Appendix 4		
	5.2	Qualitative Stochasticity Report	Summarize any observed response variability, and outline methods used to maintain reliability even without quantitative metrics.	Pg. 13-14, 17-19 Appendix 4		

Items marked with a red asterisk (*) indicate criteria that should be addressed if applicable.
